# Recent advances in cell membrane-coated porphyrin-based nanoscale MOFs for enhanced photodynamic therapy

**DOI:** 10.3389/fphar.2024.1505212

**Published:** 2024-12-04

**Authors:** Yutao Zou, Junjie Wu, Qiuyun Zhang, Jiayi Chen, Xuanxuan Luo, Yijie Qu, Rui Xia, Weiqi Wang, Xiaohua Zheng

**Affiliations:** ^1^ The People’s Hospital of Danyang, Affiliated Danyang Hospital of Nantong University, Danyang, Jiangsu, China; ^2^ School of Pharmacy, Nantong University, Nantong, Jiangsu, China; ^3^ School of Public Health, Nantong University, Nantong, Jiangsu, China

**Keywords:** photodynamic therapy, porphyrin, metal-organic frameworks, cancer cell membranes, red blood cell membranes

## Abstract

Porphyrins-based nanoscale metal-organic frameworks (nMOFs) has been widely utilized to kills tumor cells by generating cytotoxic reactive oxygen species (ROS). However, porphyrin based nMOFs (por-nMOFs) still face challenges such as rapid immune clearance and weak tumor targeting. Researchers have discovered that using a top-down biomimetic strategy, where nMOFs are coated with cell membranes, can promote long blood circulation, evade the reticuloendothelial system, and improve cancer cell targeting, thereby significantly enhancing the photodynamic therapy (PDT) effect of nMOFs. This review summarizes the recent work on different cell membranes-coated por-nMOFs for enhanced tumor PDT. This review details the changes in physicochemical properties, enhanced homotypic cancer cell-selective endocytosis, improved tumor tissue targeting, and increased cytotoxicity and effective *in vivo* tumor suppression after the nMOFs are wrapped with cell membranes. Additionally, this review compares the biological functions of various types of cell membranes, including cancer cell membranes, red blood cell membranes, aptamer-modified red blood cell membranes, and hybrid membranes from the fusion of cancer and immune cells. The review highlights the enhanced immunogenic cell death function when using hybrid membranes derived from the fusion of cancer and immune cell membranes. By summarizing the augmented PDT effects and the combined antitumor outcomes with other therapeutic modalities, this review aims to provide new insights into the biomedical applications of por-nMOFs and offer more references for the preclinical application of porphyrin-based photosensitizers.

## 1 Introduction

Malignant tumors are a type of disease characterized by extremely high mortality rates and difficulty in treatment (Ferlay et al., 2021). The relevant cancer research has prompted significant investment, including human resources, materials, and finances (Cao et al., 2021). As nanotechnology and biomedical science advance, an increasing number of treatment options are becoming available (Brigger et al., 2002; Peer et al., 2007). Compared to traditional radiotherapy, chemotherapy, and surgical treatments, photodynamic therapy is a relatively new but not yet mainstream approach (Lan et al., 2019; Chen et al., 2022; Crous and Abrahamse, 2022). However, PDT offers several advantages, such as spatiotemporal controllability, low invasiveness, low toxicity, and cost-effectiveness, making it a promising therapeutic method (Zhao et al., 2021; Di Bartolomeo et al., 2022). In PDT, light-activated photosensitizers transition from the ground state to an excited state, followed by intersystem crossing to a triplet state (Zhang et al., 2018). The triplet-state photosensitizer can then transfer energy to oxygen in tissues, generating highly reactive ^1^O_2_ that kills tumor cells (Zhao et al., 2021; Ma et al., 2022). Among the widely studied organic photosensitizers, porphyrins, bodipy dyes, methylene blue, and Cy dyes are notable, with porphyrins being particularly favored due to their excellent biocompatibility (Ethirajan et al., 2011; Ormond and Freeman, 2013; Taldaev et al., 2023). However, the hydrophobic nature and large π-conjugated system of porphyrins lead to aggregation and precipitation in aqueous environments, which necessitates the development of carrier materials to prevent this issue (Zheng et al., 2020). While micelles and dendrimers can partially mitigate aggregation, the integration of porphyrins with porous materials like MOFs has shown distinct advantages for PDT applications (Chen J. et al., 2021).

MOFs are periodic, ordered, and porous structures formed by the coordination of inorganic metal clusters with organic molecules (Horcajada et al., 2010; Wu and Yang, 2017). The modifiable structure of porphyrins allows them to be designed as ligands with four carboxyl groups, coordinating with metals such as Zr, Hf, and Fe to form MOF materials (Furukawa et al., 2013). In these MOFs, porphyrin molecules are arranged periodically and separated, which prevents intermolecular stacking and the associated decrease in ^1^O_2_ quantum yield (Chen J. et al., 2021). In comparison with conventional systems where PEG-based polymers are conjugated with porphyrin molecules to form micelles for phototherapy, it can be observed that the hydrophobic porphyrin molecules are located in the hydrophobic core and may still aggregate, leading to a decrease in the singlet oxygen quantum yield. Additionally, the conjugation process involves complex organic synthesis. On the other hand, using non-covalent interactions for encapsulation often results in low loading efficiency of the molecules. However, the high loading efficiency of porphyrin photosensitizers within the MOF matrix can be achieved (Wu and Yang, 2017). The porous nature of these materials facilitates the entry of O_2_ and the release of short-lived ROS (Park et al., 2016). This enhances the therapeutic efficacy. Research has also shown that under mildly acidic conditions, particularly in the presence of phosphates, the stability of MOFs can be compromised (Rojas et al., 2019). This will allow for the controlled release of other functional drug molecules loaded within the framework (Wang et al., 2020). This feature can be exploited for combination therapies, such as the release of L-arginine for nitric oxide (NO)-mediated gas therapy (Yang et al., 2022), TPZ for hypoxia-activated chemotherapy (Li et al., 2017a), or mitochondrial respiration inhibitors to enhance PDT by limiting oxygen consumption (Yu et al., 2023). Additionally, the surface of MOFs can be modified through non-covalent interactions to load enzymes like glucose oxidase and catalase for starvation therapy combined with PDT (Yang et al., 2022), further broadening the biomedical functions of por-nMOFs.

The growing interest in por-nMOFs as a nanoplatform is driven by these application advantages (Park et al., 2016). To improve the circulatory stability and cancer cell targeting of these nMOFs, while reducing clearance by endothelial cells, various surface modifications have been explored (Fu et al., 2022). For example, coating MOFs with polymers such as pegylation and hyaluronic acid enhances stability and promotes accumulation in tumor tissue via the enhanced permeability and retention effect (Sun et al., 2019; Fu et al., 2022). Wrapping MOFs with proteins like HSA can increase biocompatibility and reduce immune clearance (Kato et al., 2019). These modifications have shown some success in improving the therapeutic outcomes of por-nMOFs in PDT. However, to achieve even better results, researchers have turned to cell membrane coatings (Fang et al., 2018). Cancer cell membranes, red blood cell membranes, and hybrid membranes have all been used (Fang et al., 2017). Cancer cell membranes not only enhance the dispersion and circulatory stability of por-nMOFs but also facilitate active homotypic targeting and evasion of phagocytosis (Krishnan et al., 2023). Red blood cell membranes provide similar benefits, with additional aptamer modification to enhance cancer cell targeting (Luk and Zhang, 2015). Hybrid membranes, which are created by fusing cancer and immune cell membranes, can improve circulation and targeting. These membranes possess immunomodulatory functions and significantly enhance the immunogenic cell death (ICD) that is induced by photodynamic therapy (Cai et al., 2024).

**FIGURE 1:**
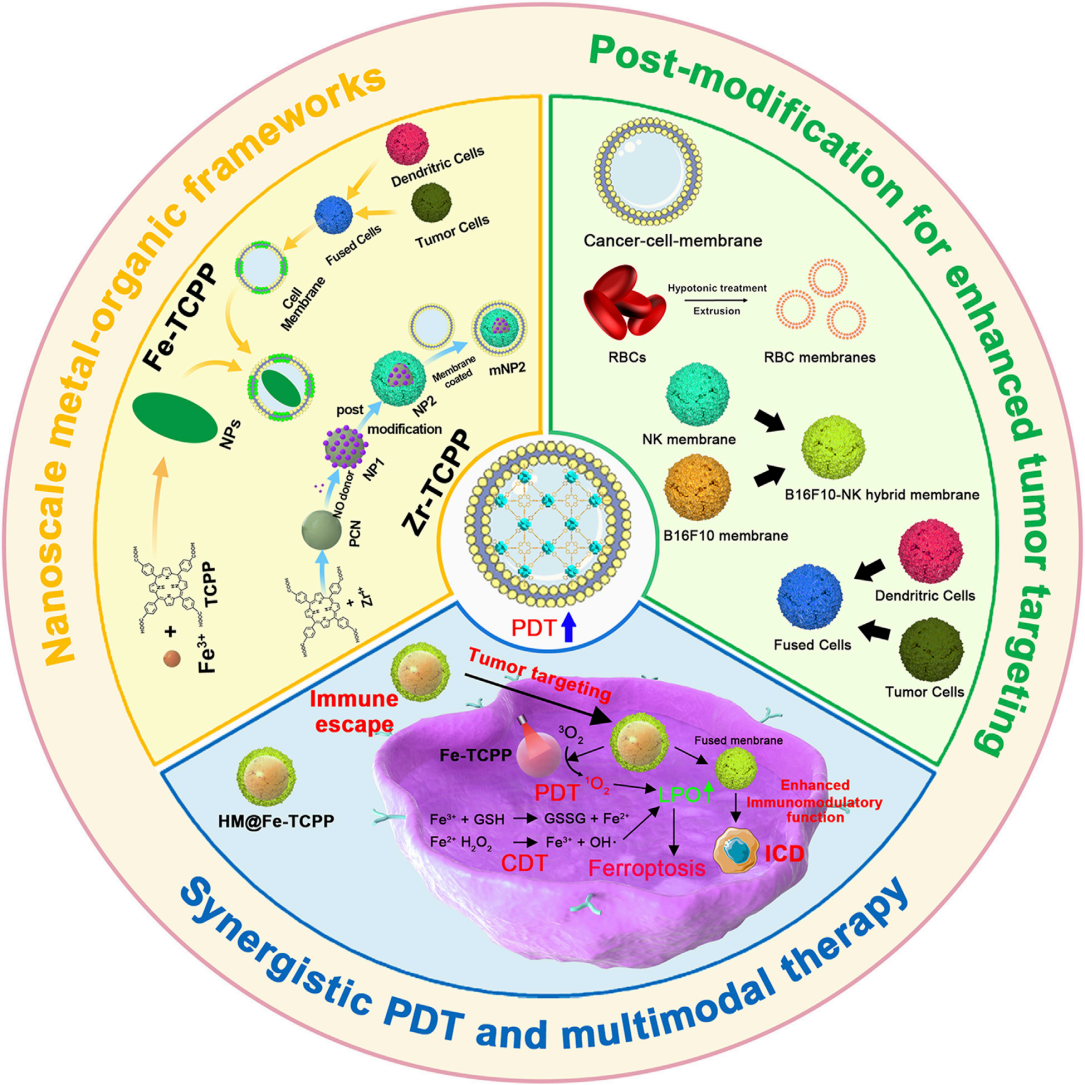
Schematic diagram of various cell membranes-coated por-nMOFs for enhanced photodynamic and multimodal therapy.

This review summarizes the advances in using various cell membrane coatings to enhance the PDT efficacy of por-nMOFs (Figure 1). We highlight the immune-evasive properties provided by these coatings, which allow for effective treatment at lower concentrations, thereby minimizing side effects. We also discuss the homotypic targeting capabilities conferred by the complex antigen profile on cancer cell membranes and the synergistic benefits of hybrid membranes in promoting ICD. By comparing the functional characteristics of different cell membranes, we aim to provide a comprehensive analysis of their respective advantages. This review compiles recent work on the synergistic mechanisms of cell membrane-coated, por-nMOFs for enhanced PDT and combination therapies against tumors (Figure 1). Through these insights, we hope to promote the further application of por-nMOFs in oncology and offer new design perspectives and reference value for the preclinical application of porphyrin photosensitizers.

## 2 PCN MOF and cell membrane acquisition procedures and mixing strategies

To highlight the specific information of cell membrane-coated MOF (CM@MOF) obtained by combining por-nMOFs with cell membranes, Table 1 details the various sizes and therapeutic mechanisms of CM@MOF materials for enhanced PDT. In this review, the designed porphyrins and MOFs are primarily PCN-structured frameworks (Table 1). Therefore, a brief description of the preparation steps for PCN MOFs, based on the literature, is provided as follows: ZrOCl_2_•8H_2_O (300 mg), TCPP (100 mg), and benzoic acid (2.8 g) are dissolved in DMF, and the mixture is stirred (100 mL, 90°C, 5 h). Subsequently, PCN-224 is collected via centrifugation at 12,000 rpm for 30 min and washed 3 times with DMF to obtain uniform-sized PCN MOF material (Li et al., 2017a).

**TABLE 1:** Cell membrane-coated porphyrin nMOFs for enhanced PDT Applications.

Material	Structure		Function/Therapeutic advantages	Ref.
	Cell membrane	CM@MOF		
4T1 CM Zr-PtTCPP	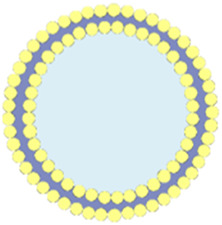	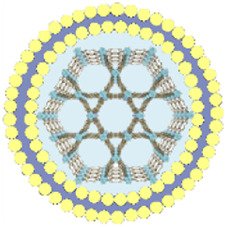	150.5 nm, −28.5 mV real time O2 sensing, homotypic targeting, immune escape, PDT	Li et al. (2018)
HeLa CM Zr-TCPP	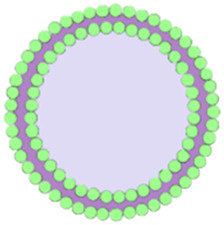	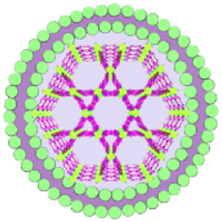	160 nm, −63.41 mV dual-mode imaging (MRI/FL), homotypic targeting, 1O2-evolving PDT	Zhang et al. (2019)
CT26 CM Zr-TCPP	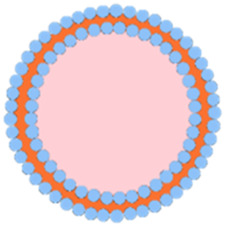	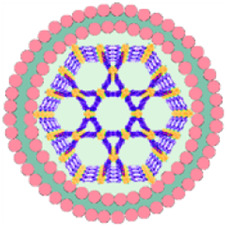	200 nm, −24 mV homotypic targeting, block ROS elimination pathway for enhanced PDT	Cheng et al. (2019b)
4T1 CM Zr-TCPP	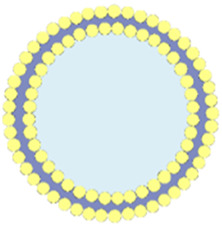	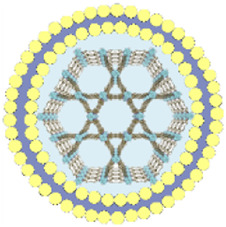	105 nm, ∼-23 mV, 660 nm, homotypic targeting, ROS-induced high NO generation, PDT + gas therapy	Wan et al. (2018)
RBCMA Cu-TCPP	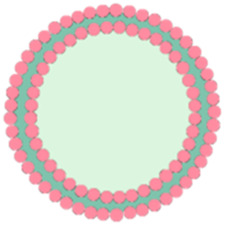	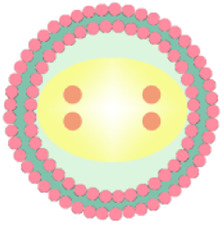	220 nm, −6.5 mV, 630 nm longer circulation, faster liver clearance, higher tumor accumulation, PDT + Chemo	Falsafi et al. (2021)
RBCMA Fe-TCPP	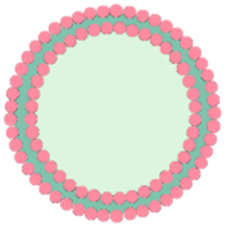	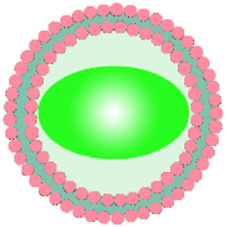	∼210 nm, −40.7 mV, longer circulation and tissue residence time, tumor-targeted, high enrichment, PDT + CDT	Zhao et al. (2020)
CT26 CM Zr-TCPP	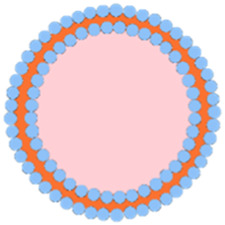	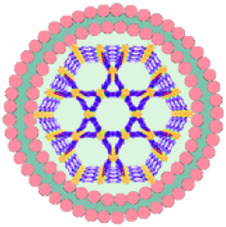	265 nm, −19.7 mV, 808 nm, 660 nm, good immune evasion, homotypic targeting, O2 generation, PDT + PTT	Cheng et al. (2019a)
4T1 CM Zr-TCPP	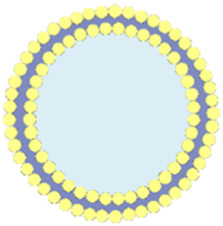	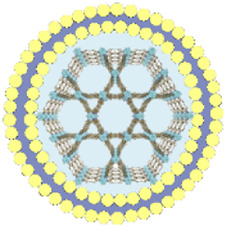	227.5 nm, −20.9 mV, 660 nm homotypic targeting, immune escape, O2 generation, PDT + ST	Li et al. (2017b)
4T1 CM Zr-TCPP	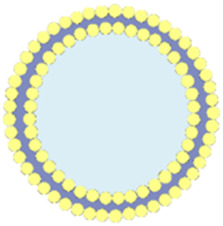	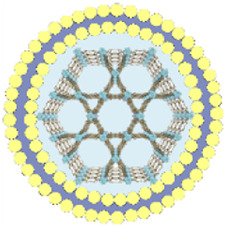	154 nm, −37.1 mV, immune escape, homotypic targeting, PDT + TPZ (hypoxia-activated bioreductive therapy)	Li et al. (2017a)
MDA-MB-231 CM Fe-TCPP	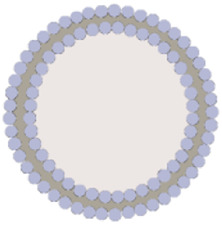	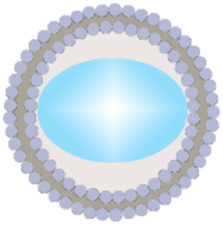	201 nm, −14.22 mV, immune escape, homotypic targeting, O2 generation, PDT + TPZ + Ferroptosis	Pan et al. (2022)
RBCM Fe-TCPP	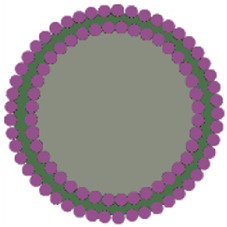	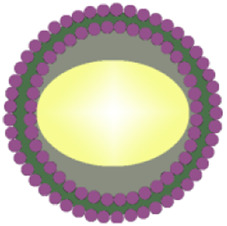	223 ± 24 nm in length, long circulation, enhanced tumor accumulation, PDT + CDT + immunotherapy	Li et al. (2022)
4T1 CM Zr-TCPP	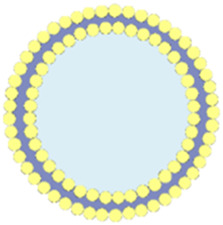	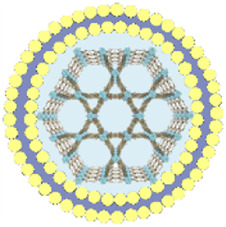	<200 nm, −36.9 mV, homotypic targeting, immune escape, NO generation, PDT + gas therapy + ST	Yang et al. (2022)
FCM1 Zr-TCPP	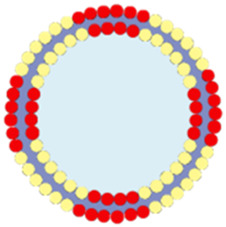	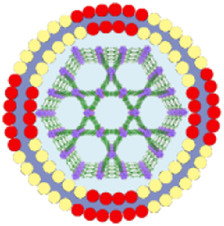	∼100 nm, −14.2 mV, homotypic targeting, immune escape, immunomodulatory functions, PDT + ICD	Gao et al. (2024)
FCM2 Fe-TCPP	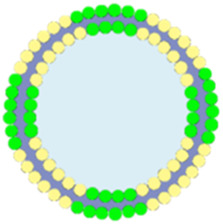	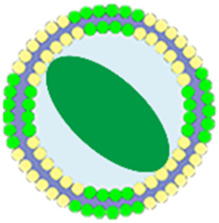	∼25 nm, ∼-7.5 mV, homotypic targeting, immune escape, immunomodulator, PDT + CDT + Ferroptosis + immunotherapy	Cai et al. (2024)

Abbreviated specification: CM, cell membrane; RBCMA, red blood cell membrane + aptamer; FCM1, hybrid membrane from NK cell and tumor cell; FCM2, hybrid membrane from DC cell and tumor cell; PDT, photodynamic therapy; chemo, chemotherapy; CDT, chemodynamic therapy; PTT, photothermal therapy; ST, starvation therapy; ICD, immunogenic cell death.

The types of cell membranes involved in this review are numerous. Different cell membranes have different sources and characteristics. For example, the 4T1 cell membrane is derived from mouse breast cancer cells, and the HeLa cell membrane is from human cervical cancer cells. The different sources of these two leads to certain differences in the components of the cell membranes, including the content of membrane proteins and the types of phospholipids on the cell membranes. Among them, different membrane proteins can affect cell-to-cell signal transduction and recognition. The phospholipid content can affect the charge distribution after the combination of the cell membrane and MOFs, thereby influencing the dispersion stability of MOFs. In addition, according to the latest data released by the International Agency for Research on Cancer, breast cancer is the most common cancer among women and has a significant impact on women’s health (Bray et al., 2024). Therefore, research and treatment of breast cancer are urgent. Given the unique homotypic targeting of 4T1 cell membranes to 4T1 cancer cells, here we take the acquisition of 4T1 cell membranes as an example. To obtain the 4T1 cell membrane, researchers first need to culture and passage 4T1 cells. The cells are then collected and resuspended in hypotonic lysis buffer containing membrane protein extraction reagents and phenylmethylsulfonyl fluoride. The cell suspension is incubated on ice for about 10–15 min. Following this, 4T1 cells undergo freeze-thaw cycles to further disrupt the cellular structure. After this process, the disrupted cells are centrifuged at 700 *g* for 10 min at 4°C to remove unbroken cells and large debris. The supernatant from the first centrifugation is then subjected to a second centrifugation at 14,000 g for 30 min to isolate the plasma membrane components. The final step involves collecting the white precipitate, which is rich in plasma membrane, and lyophilizing it for subsequent combination with nMOFs (Cheng et al., 2016; Li et al., 2017a).

To prepare CM materials, researchers can mix PCN with the cell membrane in a 1:1 mass ratio. The mixture is then extruded through a 400 nm polycarbonate porous membrane to obtain CM@PCN nanomaterials encapsulated with 4T1 cell membrane. By adjusting post-modification or preparation conditions, PCN MOF materials of varying sizes (100–300 nm) can be achieved (Li et al., 2017a). Although there are some differences in the methods of extracting different cell membranes, all extracted cell membranes exhibit negative surface potentials. After combining with MOFs, the resulting CM@MOF also displays a negative potential similar to that of the cell membranes (Table 1).

## 3 Biomimetic por-nMOFs for PDT and phosphorescence imaging of O_2_

**FIGURE 2:**
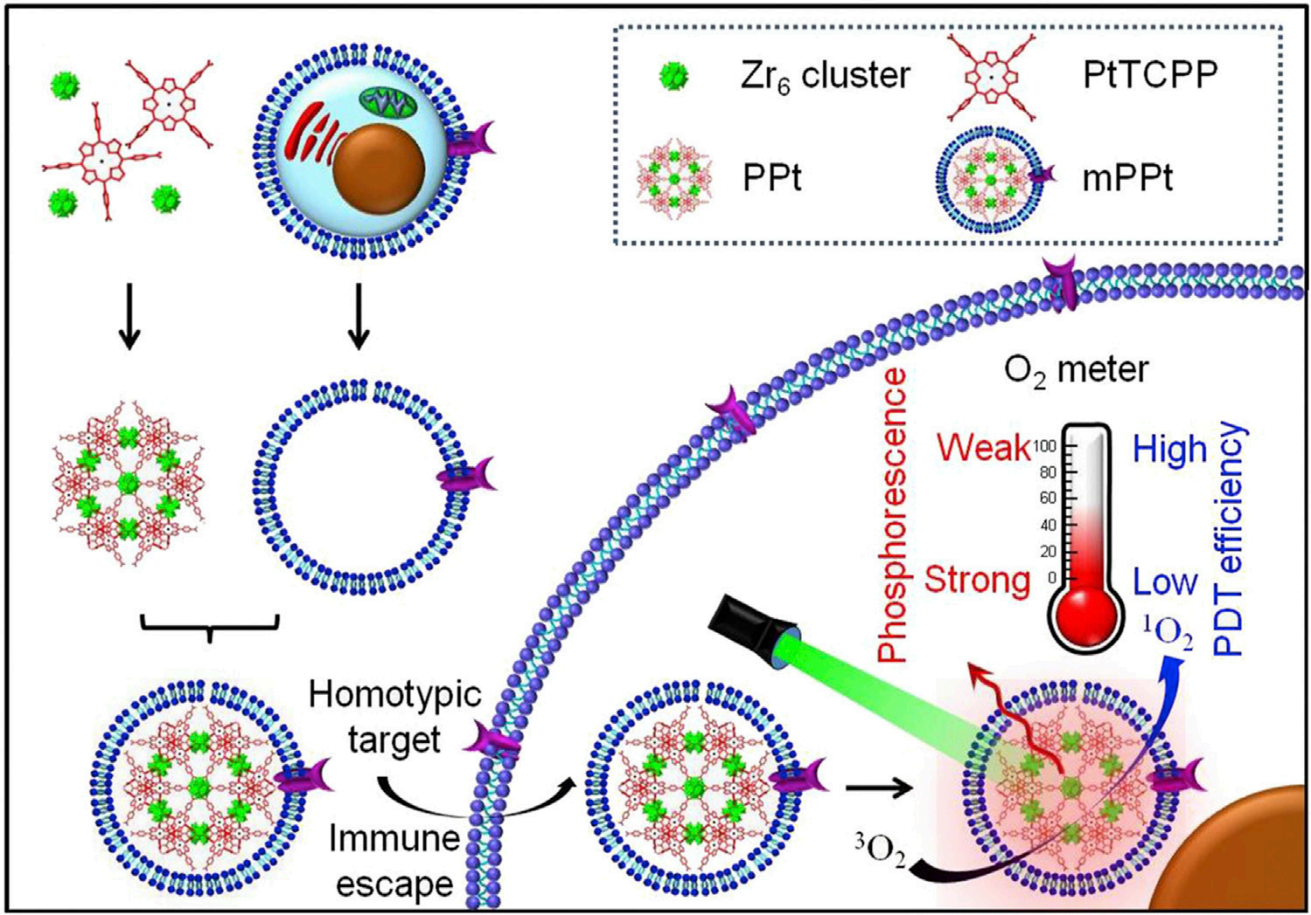
Preparation and the hypothesized mechanism of mPPt in cancer targeting and phosphorescence-guided PDT. Reproduced with permission from (Li et al., 2018). Copyright (2018), Elsevier.

Due to an uneven vascular and O_2_ supply, solid tumors are always hypoxic, which can reduce the efficacy of PDT of por-nMOFs (Zhang et al., 2019). Therefore, monitoring O_2_ concentration during PDT is crucial for predicting its therapeutic effectiveness (Li et al., 2018). Based on this concept, Li et al. designed a MOF material that can detect changes in O_2_ concentration, formed by the coordination of Zr^4+^ ion with tetracarboxylic platinum porphyrin (Li et al., 2018). The authors then coated the prepared nMOFs with cancer cell membranes to create the mPPt nanosystem (Figure 2). The presence of the cancer cell membrane provided the material with good biocompatibility, enhanced immune evasion, and improved homotypic cancer cell targeting. Moreover, the authors ingeniously utilized the phosphorescence emitted during the transition of the mPPt photosensitizer from the triplet state back to the ground state, which is quenched at high oxygen concentrations, to monitor hypoxia during PDT (Figure 2). This system cleverly integrates the PDT functionality of the cell membrane-coated por-nMOFs with real-time O_2_ concentration detection. It both elucidates the energy transfer process of the porphyrin nMOFs photosensitizer in the triplet state and highlights the critical role of hypoxia in affecting PDT outcomes. By incorporating cancer cell membranes, the authors have improved the stability of the por-nMOFs and endowed them with active homotypic targeting and immune evasion. This innovation opens new avenues and application prospects for the PDT of por-nMOFs. During PDT, monitoring oxygen concentration changes is necessary. Conventional methods like the Clark electrode method and fluorescence quenching technology have their own pros and cons (Zhang et al., 2021). The Clark electrode method is invasive and has limitations in measuring complex or moving samples for large-area and dynamic monitoring despite its high accuracy (Liebisch et al., 2020). The fluorescence quenching technology is highly sensitive, miniaturizable, portable and suitable for on-site rapid detections, but may be interfered by factors like fluorescent substance stability and environmental impurities, causing measurement result deviations (Werner et al., 2021). The phosphorescence imaging technology in this system has unique advantages, overcoming the shortcomings of the other two methods and performing well in hypoxia monitoring and for studying oxygen distribution in complex biological situations (Li et al., 2018).

## 4 Biomimetic por-nMOFs for O_2_-Evolving PDT

**FIGURE 3:**
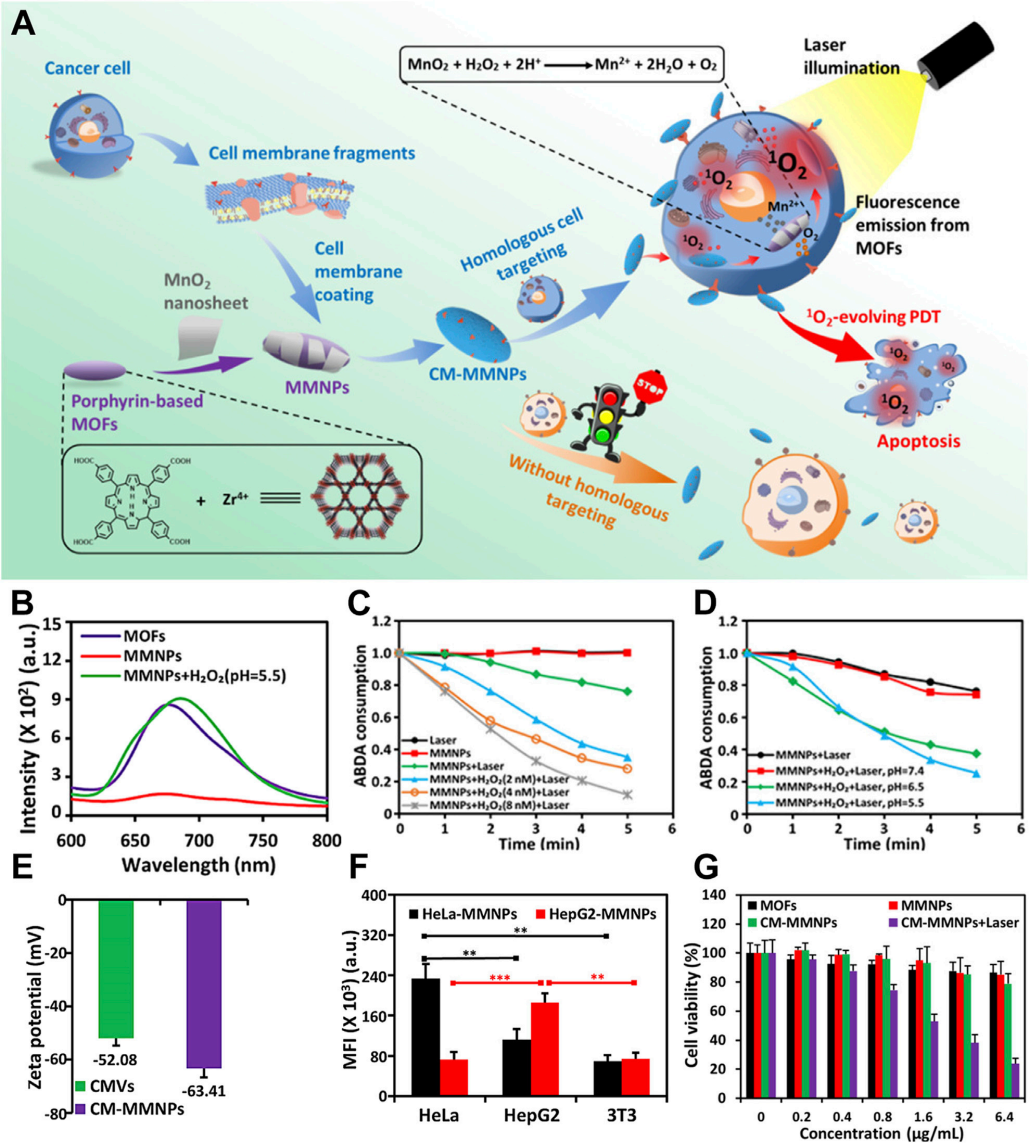
**(A)** A schematic representation of CM-MMNPs for homologous targeting, as well as for dual-mode MRI/fluorescence imaging and PDT. **(B)** Fluorescence spectra of MOFs, MMNPs, and MMNPs in the presence of H_2_O_2_. **(C)** The time-dependent consumption of ABDA under different conditions. **(D)** The time-dependent consumption of ABDA when MMNPs are exposed to laser light, with and without H_2_O_2_, at various pH levels. **(E)** Zeta potential measurements of cancer membrane vesicles (CMVs) and cancer membrane-coated magnetic multifunctional nanoparticles (CMMMNPs). **(F)** The corresponding mean intensity of red fluorescence from HeLa, HepG2, and 3T3 cells after co-incubation with HeLa cell membrane-coated CM-MMNPs. **(G)** Cytotoxicity assessment of MOFs, MMNPs, and CM-MMNPs. Reproduced with permission from (Zhang et al., 2019). Copyright (2019), American Chemical Society.

After recognizing that hypoxia can limit the efficacy of PDT using por-nMOFs, researchers have proposed strategies to alter the tumor’s hypoxic microenvironment. This may effectively enhance the performance of oxygen-dependent porphyrin-based nMOF photosensitizers. One common approach is to use inorganic materials that react with excess H_2_O_2_ within cancer cells to generate oxygen (Zhang et al., 2019). For instance, Xiao et al. developed a Zr-TCPP nMOF by coordinating tetracarboxylic porphyrin (TCPP) with metal Zr, followed by surface modification with MnO_2_ nanosheets (Zhang et al., 2019). The resulting core-shell nanostructure was then encapsulated with cell membranes to form the CM-MMNPs nanosystem. Experimental results showed that the MnO_2_ in the nanosystem could react with H_2_O_2_ under mildly acidic conditions to produce oxygen, thereby alleviating the hypoxic environment and the limitations imposed by the oxygen consumption of the por-nMOFs during PDT. Furthermore, the cell membrane coating provided the nMOFs with good dispersibility and enhanced homotypic targeting to cancer cells. This self-oxygen-generating nMOFs system presents a new strategy for treating hypoxic solid tumors with porphyrin-based photosensitizers (Figure 3A).

The diminished fluorescence of the MMNPs confirms the formation of the MnO_2_/MOF composite. Furthermore, the recovery of fluorescence upon the addition of H_2_O_2_ demonstrates the interaction mechanism between MnO_2_ and H_2_O_2_ under mildly acidic conditions (Figure 3B). To demonstrate the oxygen-generating capability of the MnO_2_, the authors used ABDA as the ^1^O_2_ scavenger to test the ability of the material to produce ^1^O_2_ under different conditions. The results showed that under mildly acidic conditions, the capacity of the por-nMOFs to generate ^1^O_2_ increased significantly with increasing H_2_O_2_ concentration (Figure 3C). Moreover, this enhancement was closely related to the weakly acidic environment (Figure 3D). In neutral conditions, the nanosystem lacks self-oxygen generation and photoreactivity, ensuring good biocompatibility. However, it shows significantly increased photoreactivity in the mildly acidic cancer cell environment. To enhance dispersibility and cancer cell targeting, the authors coated the por-nMOFs with cancer cell membranes. The zeta potential of the cancer cell membrane-coated NPs was −63.41 mV, close to the zeta potential of the cell membrane itself, confirming the successful application of the cell membrane in the system (Figure 3E). Quantitative analysis of the confocal images further supported the homotypic targeting effect of the cancer cell membranes (Figure 3F). The authors then demonstrated that the HeLa cell membrane-coated por-nMOFs system exhibited significantly higher phototoxicity under 409 nm light illumination compared to the uncoated material (Figure 3G). This system utilizes the reaction between MnO_2_ and H_2_O_2_ to provide an O_2_ source for the PDT process of por-nMOFs. Moreover, the cancer cell membrane coating significantly enhances the therapeutic effects of the por-nMOFs. This approach brings new insights into the biomedical applications of porphyrin photosensitizers.

## 5 Enhancing PDT efficacy of por-nMOFs by redox interference

In addition to the direct impact of hypoxic microenvironments on PDT efficacy, cancer cells also possess intrinsic protective mechanisms that can counteract the oxidative damage caused by ROS generated during PDT (Cheng et al., 2019b). These include antioxidants such as glutathione, small vitamin molecules, and enzymes like superoxide dismutase, catalase, peroxidases, and thioredoxin reductase. These antioxidants effectively reduce the oxidative stress induced by ROS, maintaining cellular redox balance (Cheng et al., 2019b). Since PDT relies on the generation of ROS to induce cancer cell apoptosis, its effectiveness can be significantly inhibited by these intracellular reducing agents. Therefore, disrupting the synthesis pathways of these reducing agents or inhibiting their function can enhance the efficacy of PDT. Researchers have found that the alkaloid piperlongumine (PL) increases intracellular ROS levels by inhibiting thioredoxin reductase, thereby altering the redox balance. Based on this, it was hypothesized that combining PL with por-nMOFs could enhance PDT outcomes (Cheng et al., 2019b).

**FIGURE 4:**
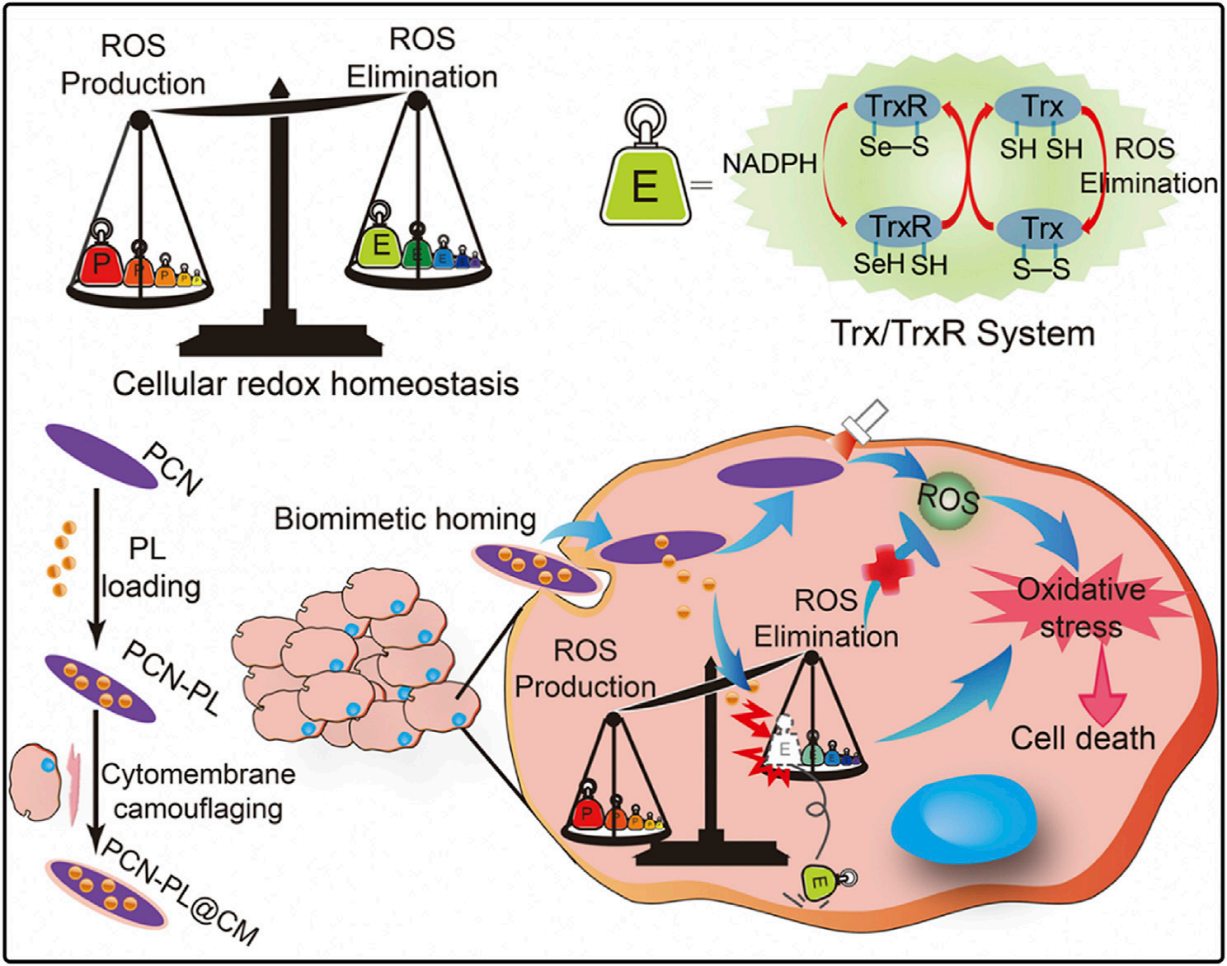
A diagrammatic representation of the strategy for disrupting redox homeostasis in cancer cells to enhance PDT. Reproduced with permission from (Cheng et al., 2019b). Copyright (2019), Elsevier.

Cheng et al. developed a PCN nMOFs by coordinating TCPP with metal Zr, which was then co-stirred and centrifuged with PL to form the PCN-PL nanosystem (Cheng et al., 2019b). This system was further coated with cell membranes through co-extrusion to create the PCN-PL@CM nanosystem. Under mildly acidic intracellular conditions, the Zr-O bonds become unstable, leading to the release of PL. Additionally, the protonation of the amine groups on PL facilitates its efficient release. Upon light exposure, the PCN-PL@CM system generates ROS, while the released PL inhibits thioredoxin reductase, thus preventing the elimination of ROS. This results in a strong oxidative stress and causes cell death (Figure 4). Cytotoxicity experiments confirmed that both PCN-PL and PCN-PL@CM effectively inhibited cell survival, exhibiting excellent phototoxicity. Subsequent animal studies showed that the PCN-PL@CM with light exposure achieved the best tumor suppression, due to the enhanced circulatory stability and homotypic cancer cell targeting provided by the cancer cell membrane coating. This system’s design shows that inhibiting intracellular antioxidants enhances the phototherapeutic function of por-nMOFs. The nMOFs’ porous structure and surface allow for loading inhibitors and coating with cancer cell membranes, improving targeting and efficacy. This approach provides valuable insights for developing advanced por-nMOFs.

## 6 Biomimetic por-nMOFs for combined PDT + gas therapy

While self-generated oxygen can effectively enhance PDT efficacy, the short-lived and limited diffusion radius of the generated ROS still pose challenges for optimal therapeutic outcomes (Cheng et al., 2016). Research has shown that certain radical species, similar to ROS, exhibit strong reactivity but have a longer half-life (∼5 s) and are more capable of diffusing within cells (40–200 μm) (Wan et al., 2018). These radicals can also react with intracellular substrates, potentially offering unexpected benefits in tumor therapy. Radicals like NO, which contain unpaired electrons, are typical examples of such biologically active species (Wan et al., 2018). In recent years, gas therapies, including CO, H_2_S, and NO, have become a focus of research for some groups (Fan et al., 2017; Zheng et al., 2021). As a green treatment with low toxicity and side effects, gas therapy has shown potential to enhance various therapeutic approaches (Zheng et al., 2021). The integration of PDT with gas therapy in nanosystems has garnered significant attention from researchers.

**FIGURE 5:**
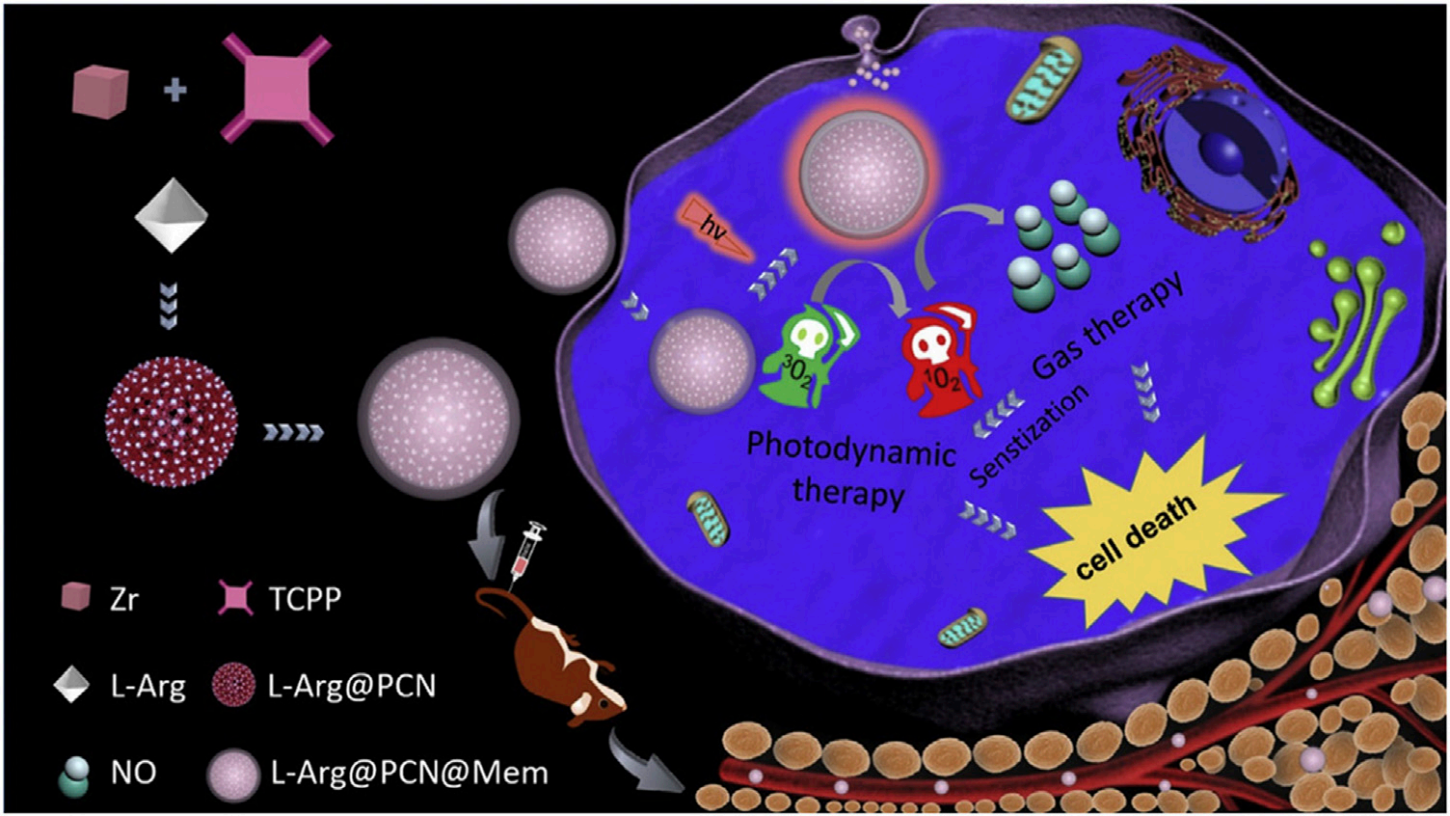
A schematic representation of the preparation of L-Arg@PCN@Mem and the lethal mechanism of gas therapy combined with sensitized PDT. Reproduced with permission from (Wan et al., 2018). Copyright (2018), Elsevier.

NO exhibits dose-dependent biological effects; at higher concentrations, it acts as a tumor suppressor, inducing cancer cell apoptosis (Wan et al., 2018). At lower concentrations, it modulates P-glycoprotein activity, helping to overcome multidrug resistance in cancer cells. Therefore, the design and development of nano-drug systems that combine NO gas with PDT may provide new avenues for enhancing PDT efficacy (Wan et al., 2018). The porous structure of por-nMOFs offers excellent drug-loading capabilities, enabling the incorporation of NO donor molecules, such as L-arginine. Based on this principle, Wan et al. prepared a PCN MOF by coordinating TCPP with Zr. They then loaded the MOF with L-arginine and subsequently encapsulated it with cancer cell membranes, resulting in the L-Arg@PCN@Mem nanoplatform (Figure 5) (Wan et al., 2018). Under light exposure, this nanoplatform generates ^1^O_2_, which oxidizes L-arginine to produce large amounts of NO. High concentrations of NO can induce cytotoxicity, thereby achieving a combined therapeutic effect of PDT and NO-mediated gas therapy (Figure 5). The homotypic targeting capability of the cancer cell membrane enhanced the efficient inhibition of L-Arg@PCN@Mem + L on 4T1 cell proliferation. Based on the analysis of the experimental results, L-Arg alone under light exposure barely causes cell death, indicating the good biocompatibility of L-Arg and its advantage as a NO donor. Compared to PCN under light exposure, the cytotoxicity of the L-Arg-loaded L-Arg@PCN nanosystem is further increased (cell viability decreases from approximately 70% to around 62%). Notably, when encapsulated with a cell membrane, L-Arg@PCN@MeM under light exposure leads to a cell viability of approximately 12.5%, while PCN@MeM without L-Arg results in a cell viability of 37.5%. These comparative experimental results suggest that cell membrane encapsulation is more conducive to enhancing the cytotoxic effect of NO. This is because the enhanced endocytosis promoted by the cell membrane facilitates an increase in the intracellular concentration of L-Arg, leading to a higher release of NO, which is more effective in exerting cytotoxic effects and synergizing with PDT. This system uses 4T1 cell membranes to encapsulate por-nMOFs, enhancing stability and targeting of cancer cells. This approach advances antitumor applications of por-nMOFs.

## 7 RBC membrane-coated por-nMOFs for enhanced PDT

**FIGURE 6:**
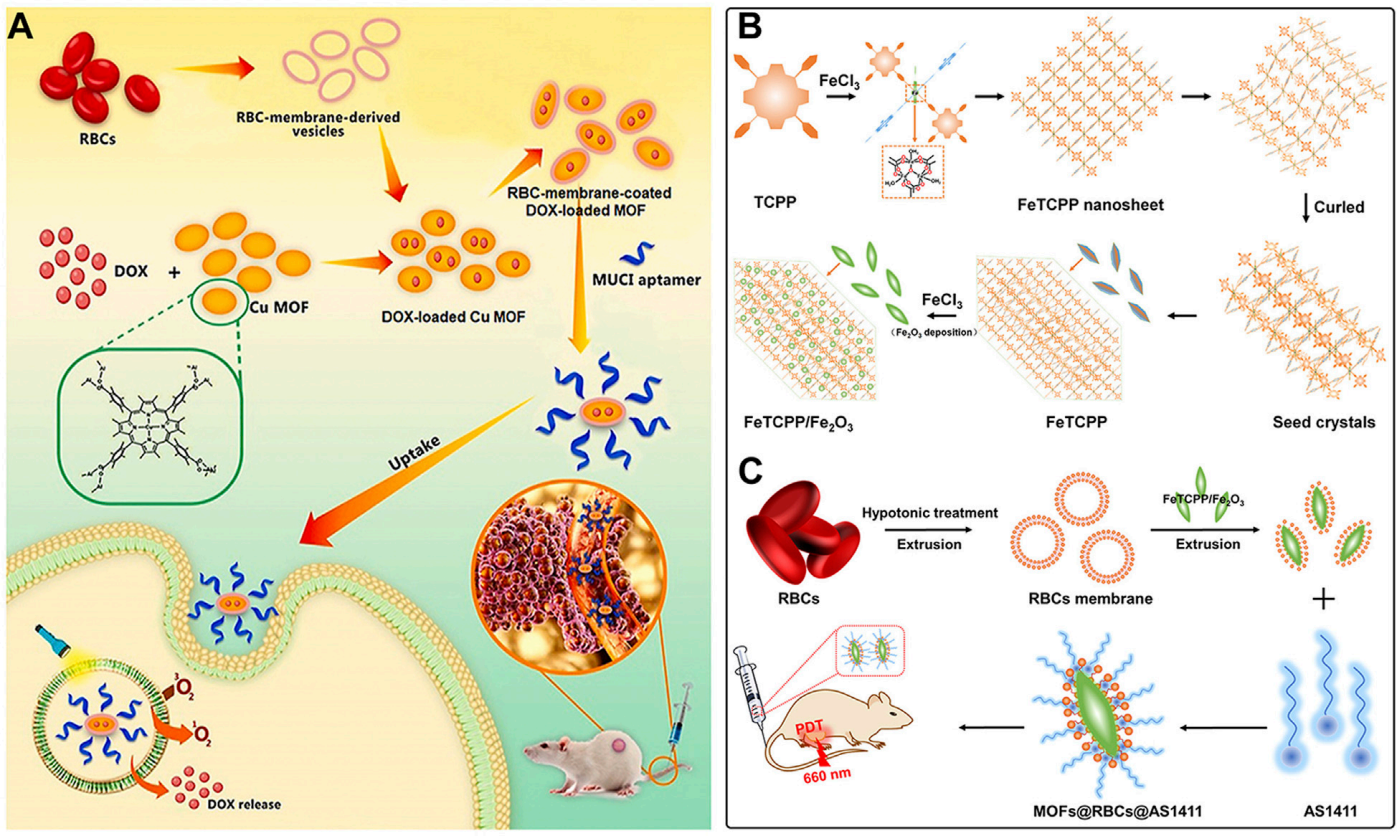
**(A)** The preparation of RBC-membrane-coated DOX-loaded MOF for PDT and chemotherapy. Reproduced with permission from (Falsafi et al., 2021). Copyright (2021), Elsevier. **(B)** Synthesis process of FeTCPP and the FeTCPP/Fe_2_O_3_ MOF nanorice; **(C)** Illustration of the MOFs@RBCs@AS1411 nanorice for targeted PDT. Reproduced with permission from (Zhao et al., 2020). Copyright (2020), American Chemical Society.

Apart from cancer cell membranes, researchers have found that using aptamer-modified red blood cell membranes (RBCM) for surface modification are same effective (Falsafi et al., 2021). The RBCM encapsulation not only improves the stability of the materials, prolongs their circulation time in the bloodstream, and reduces phagocytosis by macrophages, but also confers selectivity towards a variety of cancer cells (Falsafi et al., 2021). Based on this concept, Monireh Falsafi et al. extracted RBC membranes and used them to encapsulate MOF materials pre-loaded with the chemotherapeutic drug DOX (Falsafi et al., 2021). The MOF was prepared by coordinating a porphyrin photosensitizer with metal Cu, resulting in the RBC-MOF@DOX nanosystem. The RBCM was then functionalized with carboxylated aptamers (MUC1), which have the specific ability to recognize tumor cells. Subsequent research demonstrated that the fabricated nanosystem could simultaneously target 4T1 and MCF7 breast cancer cells, achieving a combined therapy of chemotherapy and PDT, effectively inhibiting cancer cell proliferation (Figure 6A).

In addition, Zhao et al. used water and chloroform as immiscible phases and employed a liquid-liquid diffusion method to synthesize FeTCPP nMOFs and an iron oxide (Fe_2_O_3_) loaded nanosystem (FeTCPP/Fe_2_O_3_) at the interface between the two phases (Zhao et al., 2020). The authors then isolated RBCs and modified their surface with the AS1411 aptamer. Subsequently, they combined the RBC membrane with the nMOFs material to create the MOFs@RBCs@AS1411 nanosystem, which exhibited enhanced PDT effects under 660 nm laser irradiation (Figures 6B, C). In this system, the authors controlled the ratio of porphyrin molecules to ferric ions to produce a Fe-TCPP MOF and Fe_2_O_3_ composite, enabling a combined CDT and PDT mechanism. Encapsulation with aptamer-modified RBC membranes significantly enhanced the circulation time and tumor accumulation of the nanomaterial in the bloodstream. This system, through the encapsulation of nMOFs with RBCM and their surface modification with cancer-specific aptamers, has shown a significant enhancement in the phototherapeutic function of por-nMOFs. The results indicate the potential of cell membrane-coated por-nMOFs for preclinical research applications. It should be noted that although the nMOFs coated with red blood cell membranes have shown significant advantages in phototherapy, the potential safety hazards that some foreign RBCM may bring to the body still cannot be ignored (Villa et al., 2016; Liu et al., 2020). Before using xenogeneic RBCM to coat nanomedicines, the surface proteins of RBCM should be detected to prevent variation (Zheng and Xiao, 2022). Meanwhile, for blood donors, blood compatibility tests and infectious disease screenings are also required, which are of great significance for preventing potential immune responses (Zheng and Xiao, 2022).

## 8 Biomimetic por-nMOFs for combined PDT + PTT

In addition to the combination with gas therapy, chemotherapy, and CDT, the porosity and modifiability of por-nMOFs also provide an advantage for integrating photothermal functionalities. Researchers have found that the efficacy of standalone PDT is often limited by the presence of hypoxic solid tumors, while standalone photothermal therapy (PTT) may cause damage to surrounding tissues due to excessive heat (Cheng et al., 2019a; Su et al., 2019; Yu et al., 2019). Moreover, upregulated heat shock proteins within cells can counteract the effects of PTT (Chen G. et al., 2021; Liu et al., 2022). Therefore, some research groups have sought to develop nanoplatforms that combine the therapeutic effects of PDT and PTT (Cheng et al., 2019a). The multifunctionality of nMOFs makes such a system possible.

**FIGURE 7:**
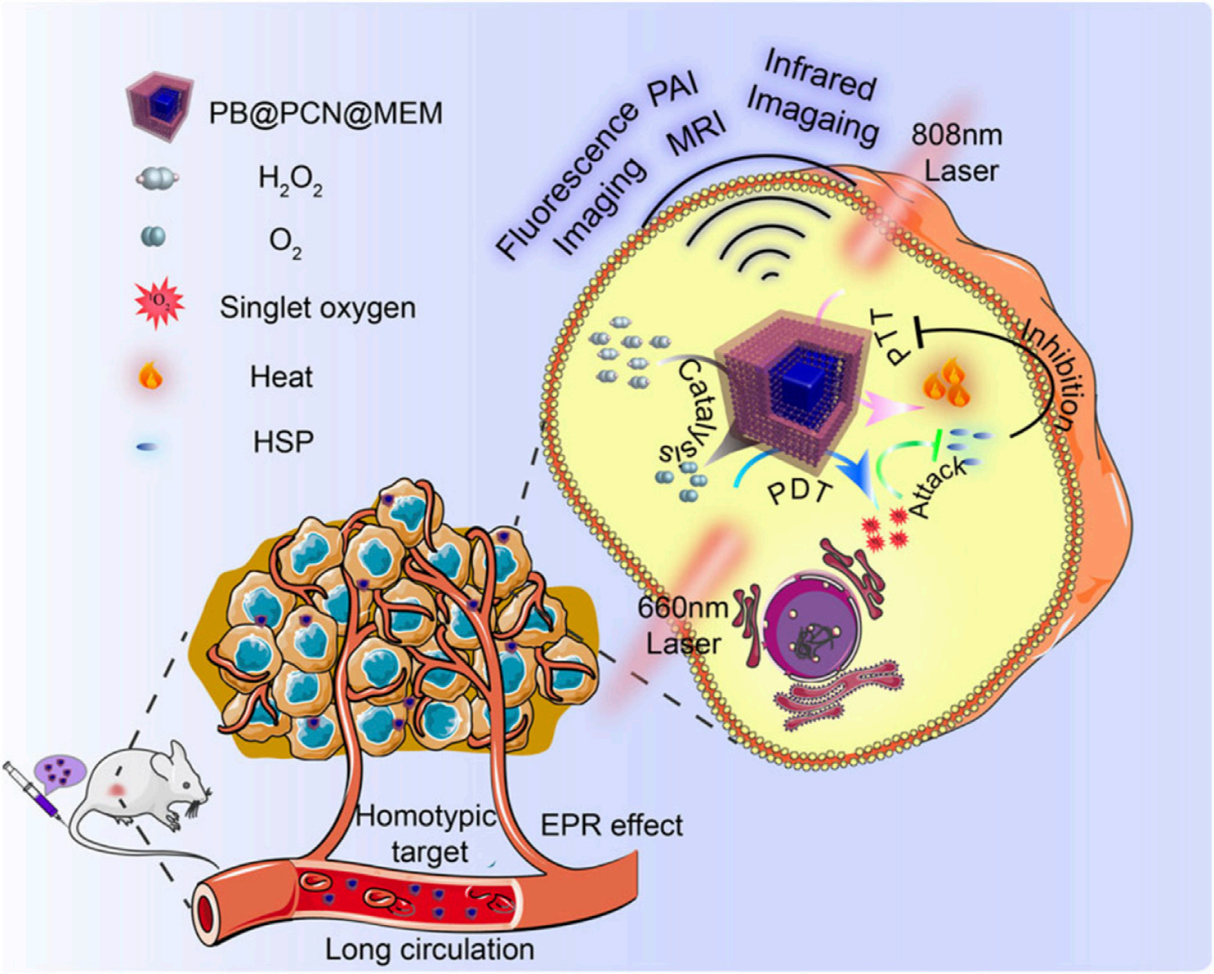
A schematic representation of the PB@PCN@MEM system for PTT and PDT. Reproduced with permission from (Cheng et al., 2019a). Copyright (2019), Nature Publishing Group.

For example, Cheng et al. first synthesized Prussian Blue (PB) NPs with the assistance of polyvinylpyrrolidone (PVP) (Cheng et al., 2019a). They then added different concentrations of TCPP and zirconium ions to react and form a series of PB/PCN nanocomposites with varying shell thicknesses. The authors selected the optimal PB/PCN composite and demonstrated its ability to effectively inhibit the proliferation of CT26 cancer cells in both cellular and animal models (Figure 7). Through rational design, this system integrates por-nMOFs onto the surface of Prussian blue. It leverages the catalase-like activity of PB to decompose H_2_O_2_ and generate O_2_, thereby improving the hypoxic microenvironment and enhancing the efficacy of PDT. On the other hand, it utilizes 808 nm laser irradiation to achieve a combined PDT and PTT effect. The combination of photothermal therapy and photodynamic therapy is a highly effective strategy against tumor proliferation. The application of PTT, on the one hand, can avoid the limitation of hypoxia on PDT (Zheng et al., 2020). On the other hand, photothermal therapy promotes local oxygen transport, which is beneficial to the type-II PDT mechanism that depends on oxygen concentration to generate singlet oxygen for treatment. However, it should be noted that in the complex physiological environment *in vivo*, the photothermal conversion efficiency of Prussian blue nanoparticles may be affected. Prussian blue nanoparticles may not be degraded in a timely manner *in vivo*. If the nanoparticles cannot be effectively degraded in the body, they may gradually accumulate in tissues or organs. This may cause potential toxicity, and more experimental data are required to verify the biomedical applications of Prussian blue. Additionally, the encapsulation with CT26 cancer cell membranes enhances the circulation stability and immune evasion of the material. The cell membrane coating provides the material with enhanced homotypic cancer cell targeting, offering better assurance for the multifunctional therapy of por-nMOFs.

## 9 Biomimetic por-nMOFs for PDT + starvation therapy

The application of nMOFs in the biomedical field is extremely broad. In addition to using their porous structure to load small molecules for combined gas therapy, chemotherapy, and other treatments, nMOFs can also interact electrostatically with larger biomolecules, such as proteins, due to their high specific surface area (Li et al., 2017b). This allows for the integration of por-nMOFs-mediated PDT with various biological enzymes for combined therapeutic effects.

**FIGURE 8:**
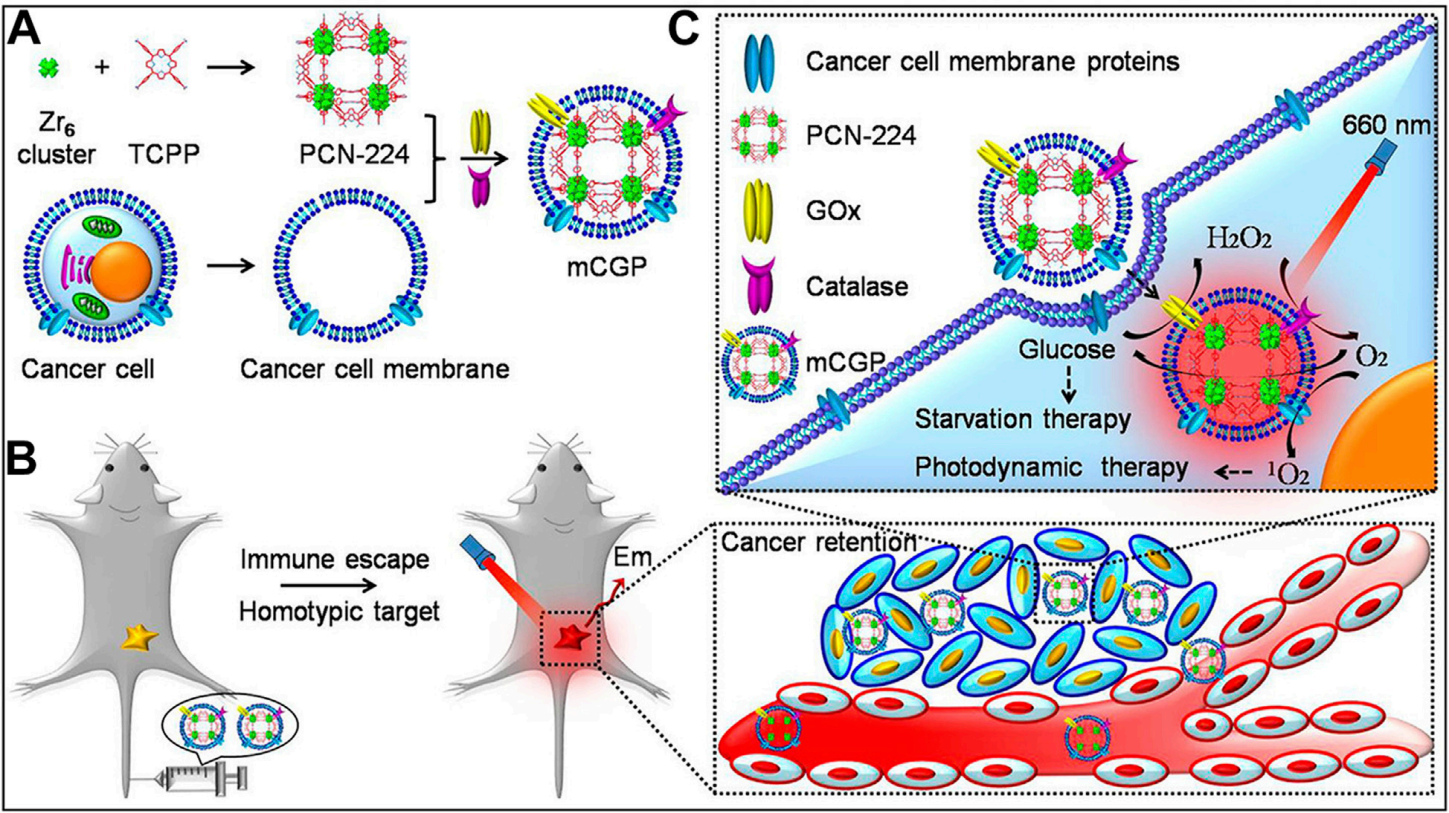
**(A)** The preparation steps for mCGP. **(B)** The immune evasion and homotypic targeting capabilities of mCGP. **(C)** Cascade reactions that enhance the synergistic effects of mCGP, cutting off the glucose supply to cancer cells for starvation therapy and promoting ^1^O_2_ generation for PDT. Reproduced with permission from (Li et al., 2017b). Copyright (2017), American Chemical Society.

Based on this concept, Li et al. used a conventional dissolution method to synthesize PCN-224 MOF materials from TCPP and zirconium salts (Li et al., 2017b). They then loaded glucose oxidase (GOx) and catalase (CAT) onto the MOF surface through electrostatic interactions, achieving a loading efficiency of up to 13.5%. The material was subsequently encapsulated with 4T1 cancer cell membranes to form the mCGP nanocomposite system. Subsequent cellular and animal experiments demonstrated that mCGP could achieve enhanced PDT through the action of catalase and starvation therapy via glucose oxidase (Figures 8A–C). The successful design of this system further demonstrates the multifunctionality of por-nMOFs. The large specific surface area of these nMOFs enables the integration of multiple therapeutic functions through the combination with various enzymes. Additionally, the encapsulation with 4T1 cancer cell membranes significantly enhances the homotypic targeting of the por-nMOFs towards 4T1 cancer cells. The excellent antitumor efficacy of this system provides new insights for the preclinical application of por-nMOFs.

## 10 Biomimetic por-nMOFs for PDT and TPZ chemotherapy

**FIGURE 9:**
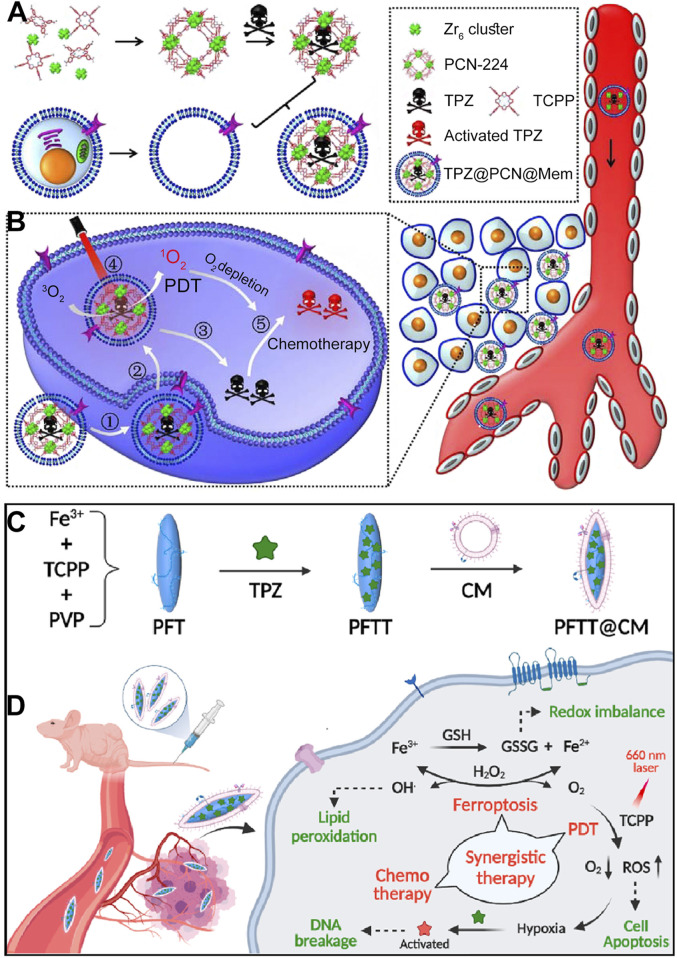
**(A)** Step-by-step assembly process of the cancer cell membrane-coated biomimetic nanoplatform TPZ@PCN@Mem. **(B)** Diagrammatic representation of the multifunctional nanoplatform, which exhibits enhanced tumor accumulation for targeted PDT and hypoxia-activated bioreductive therapy. Reproduced with permission from (Li et al., 2017a). Copyright (2017), Elsevier. **(C)** Fabrication process of the cell membrane-coated nanocomposites PFTT@CM. **(D)** Illustration of the synergistic therapeutic effects of PFTT@CM, which integrates ferroptosis, PDT and hypoxia-activated chemotherapy. Reproduced with permission from (Pan et al., 2022). Copyright (2022), Elsevier.

In addition to using catalase to generate O_2_ from H_2_O_2_ to improve the hypoxic microenvironment and enhance PDT efficacy, another approach involves incorporating hypoxia-sensitive chemotherapeutic drugs to achieve a combined PDT and chemotherapy mechanism mediated by por-nMOFs (Zhang et al., 2024). For example, Li et al. synthesized PCN-224 MOF using zirconium ions and TCPP. They then co-stirred the MOF with tirapazamine (TPZ) molecules, followed by centrifugation to obtain TPZ-loaded TPZ@PCN composites (Li et al., 2017a). To enhance the circulation stability, immune evasion, and cancer cell targeting of the MOF material, the authors encapsulated the TPZ@PCN composites with 4T1 cancer cell membranes, resulting in the TPZ@PCN@Mem nanocomposite system (Figure 9A). Subsequently, at both cellular and animal levels, the authors demonstrated that the TPZ@PCN@Mem nanoplatform could exert excellent PDT effects under light exposure. The PDT process exacerbated the hypoxic microenvironment, and intracellular phosphates promoted the release of TPZ molecules from the nMOF. In the hypoxic microenvironment, TPZ is reduced to highly toxic free radicals, thereby exerting its chemotherapeutic function. The results showed that the 4T1 cancer cell membrane-encapsulated por-nMOFs could achieve a combined effect of PDT and hypoxia-activated chemotherapy (Figure 9B). The successful design of this system brings new hope for the phototherapeutic application in hypoxic solid tumors and aids in the precise and effective eradication of hypoxic solid tumors by por-nMOFs.

In addition to the common PCN framework MOFs formed by the coordination of Zr^4+^ with TCPP, Fe^3+^ can also coordinate with TCPP to form a spindle-shaped nMOF material. Researchers have found that the Fe^3+^ in Fe-TCPP MOFs can interact with excess GSH within cancer cells (Pan et al., 2022). This interaction not only reduces GSH levels, which is beneficial for ROS-mediated cancer therapy, but also generates Fe^2+^ ions. Under mildly acidic conditions, Fe^2+^ ions could react with H_2_O_2_ to form highly oxidative hydroxyl radicals (Pan et al., 2022). These radicals oxidize lipids, producing more lipid peroxidation products and inducing ferroptosis (Zou et al., 2024). Ferroptosis is a widely-studied approach for disease treatment (Wang et al., 2022; Li Z. et al., 2023; Mao et al., 2023), and the combination of ferroptosis and phototherapy may lead to unexpected results.

For example, Pan et al. synthesized PFT NPs (Fe-TCPP MOF) under the assistance of PVP (Pan et al., 2022). They then added TPZ molecules, stirred, and centrifuged the mixture to obtain PFTT NPs loaded with the chemotherapeutic drug TPZ. To further enhance the targeting of the nanomaterials towards cancer cells, the authors coated the PFTT NPs with breast cancer cell (MDA-MB-231) membranes, resulting in the PFTT@CM nanosystem (Figure 9C). Subsequently, at the cellular level, the authors demonstrated the multifunctional combined therapeutic effects of the PFTT@CM nanosystem. The breast cancer cell membrane coating facilitated the targeted endocytosis of the nanomaterials by breast cancer cells. Upon internalization, the presence of phosphates led to the release of TPZ. Under light exposure, the porphyrin photosensitizer generated ^1^O_2_, exerting PDT effects. This process further exacerbated the hypoxic microenvironment, promoting the conversion of TPZ into its radical form, which significantly enhanced cytotoxicity. Additionally, the Fe^3+^ ions in the MOF structure could interact with excess intracellular GSH and H_2_O_2_, generating highly oxidative hydroxyl radicals and forming a significant amount of lipid peroxidation products. This ultimately achieved a combined therapeutic effect involving porphyrin-mediated PDT, TPZ-mediated chemotherapy, and Fe-mediated ferroptosis (Figure 9D). The encapsulation with breast cancer cell membranes enhanced the circulation stability of the porphyrin-based nMOFs materials and their homotypic targeting towards cancer cells, providing a basis and possibility for improved therapeutic outcomes. This successful design demonstrates the potential of combining multiple therapeutic modalities using a single nanoplatform, and it highlights the importance of biomimetic strategies for enhancing the selectivity and efficacy of cancer treatments. The use of cancer cell membranes for surface modification not only improves the biocompatibility and targeting of the nanomaterials but also provides a promising approach for the precise and effective treatment of solid tumors, particularly those with a hypoxic microenvironment.

## 11 Biomimetic por-nMOFs for combined PDT + CDT + ICD

**FIGURE 10:**
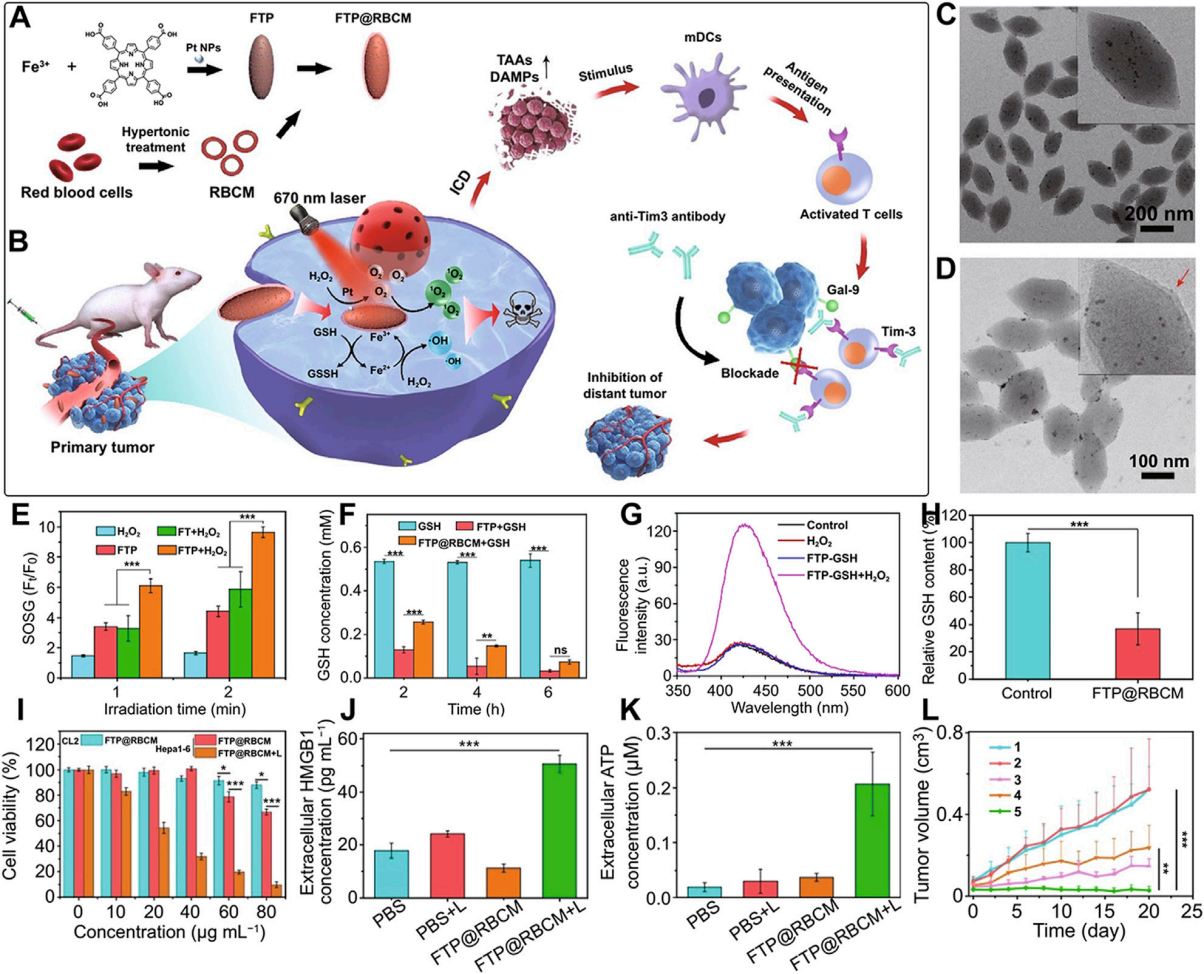
**(A)** Synthesis of FTP@RBCM. **(B)** Mechanisms of the combined immunotherapy involving radical therapy and Tim-3 checkpoint blockade. **(C)** TEM image of FTP. **(D)** TEM image of FTP@RBCM. **(E)** Relative fluorescence intensity of SOSG. **(F)** GSH concentration following various treatments. **(G)** Fluorescence intensity of the solution containing TPA under different treatment conditions. **(H)** Relative GSH content in Hep3B cells after various treatments. **(I)** Viability of CL2 and Hepa1-6 cells. **(J)** Extracellular levels of high-mobility group box 1 (HMGB1). **(K)** Extracellular HMGB1 levels. **(L)** Tumor growth curves in mice. Group 1: PBS; Group 2: PBS + Light; Group 3: FT@RBCM + Light; Group 4: FTP@RBCM; Group 5: FTP@RBCM + Light. Reproduced with permission from (Li et al., 2022). Copyright (2022), Springer Nature.

nMOFs can not only load organic small molecules but also inorganic materials, with different strategies employed for each. For small molecules like TPZ, the porous structure of nMOFs can be utilized for loading after the nMOFs is formed. In addition, some inorganic metal NPs can be doped into the nMOFs during its synthesis, typically requiring the metal NPs to be of a smaller size (Li et al., 2022). For example, Yao et al. used a solvothermal one-pot method to synthesize Fe-TCPP MOFs, doping them with 3 nm Pt NPs to create FTP NPs, a nanocomposite material loaded with inorganic metal (Li et al., 2022). The undoped MOF material was labeled as FT NPs. To enhance the cancer cell targeting of the material, the authors extracted red blood cell membranes (RBCM) to encapsulate the FTP, resulting in the FTP@RBCM nanosystem (Figure 10A). At the cellular level, the authors demonstrated that the Pt NPs in the FTP@RBCM nanosystem had catalase-like activity. They could catalyze the excess H_2_O_2_ within cancer cells to produce O_2_. As a result, this relieved the hypoxic microenvironment and enhanced the PDT efficacy. Additionally, the iron within the MOFs is capable of interacting with the excessive intracellular GSH, thereby reducing the levels of reducing agents. The generated Fe^2+^ ions could then react with intracellular H_2_O_2_, initiating a chemodynamic process to form highly oxidative hydroxyl radicals. The combination of a large number of ·OH and the ^1^O_2_ produced under light exposure induced severe oxidative stress, leading to apoptosis. Furthermore, the authors found that high concentrations of ROS also triggered acute local inflammation and immunogenic cancer cell death (Figure 10B).

TEM results confirmed the spindle-shaped structure of the synthesized Fe-TCPP (Figure 10C) and showed a clear core-shell structure after RBCM encapsulation (Figure 10D). To demonstrate the catalase-like function of the Pt NPs, the authors mixed FT NPs and FTP NPs with H_2_O_2_ and used SOSG as a ^1^O_2_ sensor to detect the photosensitizer’s ability to generate ^1^O_2_ under light exposure. The results showed that the FTP material containing Pt NPs had the strongest ^1^O_2_ generation capability, confirming that the Pt NPs effectively catalyzed H_2_O_2_ to produce O_2_, thus enhancing the PDT process (Figure 10E). Additionally, comparative experiments showed that FTP NPs could utilize Fe^3+^ to reduce the concentration of intracellular GSH (Figure 10F). Using terephthalic acid (TPA) as a probe, which becomes hydroxylated and exhibits enhanced fluorescence upon reaction with ·OH, the authors detected changes in ·OH concentration by measuring fluorescence intensity. The results indicated that Fe^2+^ generated from the interaction between Fe^3+^ and GSH could effectively react with intracellular H_2_O_2_ to produce ·OH (Figure 10G). Intracellularly, the authors also observed that FTP@RBCM could effectively reduce GSH levels, facilitating enhanced ROS-mediated therapy (Figure 10H). Cell toxicity experiments showed that the RBCM-coated nanosystem could recognize normal cells and avoid being endocytosed. However, it was efficiently taken up by Hepa1-6 cancer cells. Under light exposure, it significantly inhibited the proliferation of these cancer cells (Figure 10I). To verify that the large amount of ROS generated by the nanomaterials under light exposure could trigger an immune response and induce ICD, the authors measured intracellular immune factors, and the results were consistent with expectations. Subsequently, the authors assessed several ICD-related factors. The experimental results showed that, compared to other control groups, the FTP@RBCM + L group had the least HMGB1 signal in the cell nucleus, indicating that many HMGB1 molecules were released, with the highest extracellular HMGB1 concentration detected (Figure 10J). Additionally, as a key feature of ICD known for recruiting immune cells and triggering inflammatory effects, ATP was most prominently secreted by cells treated with FTP@RBCM + L (Figure 10K). The high ATP levels detected indicated a significant ICD effect. At the animal level, the authors also demonstrated that the FTP@RBCM + L treatment group had the best inhibitory effect on Hepa1-6 tumor proliferation (Figure 10L).

This system utilizes por-nMOFs to load inorganic Pt NPs, and enhances cancer cell targeting through RBCM encapsulation. The system achieves enhanced PDT efficacy due to the catalase-like activity of Pt NPs, which generates O_2_, and the chemodynamic mechanism mediated by Fe, which produces ·OH. The combined strong oxidative stress from ^1^O_2_ and ·OH triggers an ICD effect. The successful design of this nanosystem provides a new reference for the antitumor applications of por-nMOFs in conjunction with cell membranes. In this system, utilizing oxidative stress to induce the ICD effect for cancer cell inhibition is a highly effective and innovative idea. However, immunosuppression within the tumor microenvironment may affect the outcome of immunogenic cell death. Appropriately adopting other combined treatment modalities might further enhance the ICD effect. For example, using immune checkpoint inhibitors may strengthen the ability of the immune system to kill tumor cells, thereby improving the effect of ICD (Duan et al., 2019). Alternatively, modifying CAR-T cells can overcome the immunosuppression in the tumor microenvironment and enhance the effect of its combination with ICD-inducing therapies (Li H. et al., 2023). These are all topics worthy of further research.

## 12 Biomimetic por-nMOFs for combined PDT + gas therapy + starvation therapy

**FIGURE 11:**
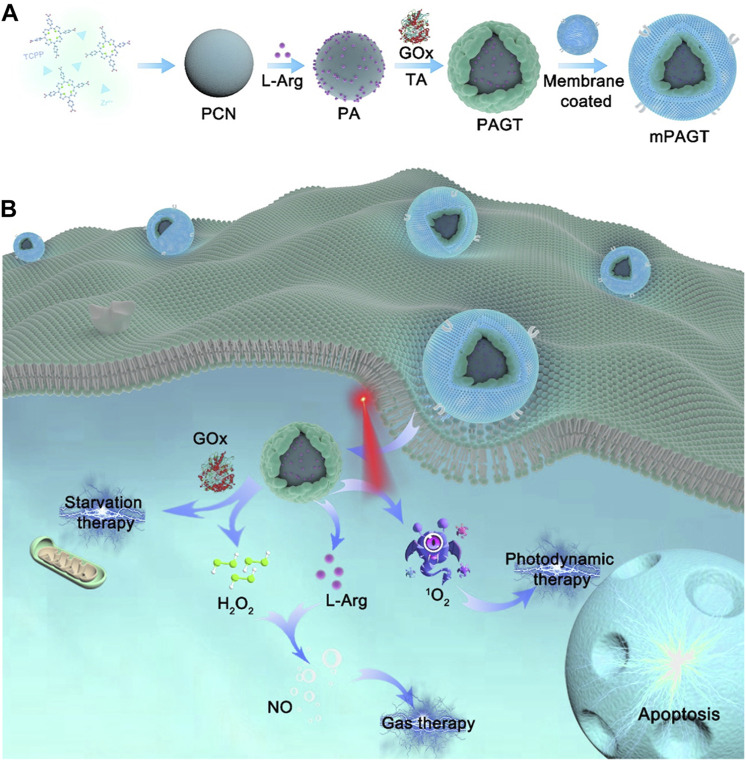
**(A)** The fabrication process of mPAGT NPs. **(B)** The mechanism of the lethal effects achieved through the catalytic cascades-enhanced synergistic therapy. Reproduced with permission from (Yang et al., 2022). Copyright (2022), Elsevier.

Exploiting the porous nature and surface modifiability of nMOFs, it is possible to simultaneously load both small molecules and biomacromolecules (Yang et al., 2022). For example, Yang et al. used Zr and TCPP as precursors to synthesize PCN MOF via a solvothermal method (Yang et al., 2022). They then loaded L-arginine, which can be oxidized to release NO, into the pores of the nMOFs, resulting in PA NPs. Subsequently, the authors mixed polyphenolic tannic acid, rich in hydroxyl groups, with the PA NPs to create a hydroxyl-rich environment on the nMOFs surface. By leveraging the interactions between these hydroxyl groups and proteins, they incorporated glucose oxidase (GOx) to ultimately form PAGT NPs (Figure 11A). To enhance the circulation stability and tumor cell targeting of the prepared PAGT NPs, the authors further coated the PAGT NPs with 4T1 cancer cell membranes through ultrasonication, creating the mPAGT nanosystem. At the cellular level, the authors demonstrated that under light exposure, this nanosystem could achieve a combined therapeutic effect. The effect involved porphyrin-mediated PDT, NO release from L-arginine, and starvation therapy mediated by GOx. The 4T1 cell membrane coating significantly enhanced the inhibitory effect of the material on 4T1 cells, highlighting the advantage of the homotypic targeting capability inherent in cancer cell membranes (Figure 11B). This system leverages the unique properties of nMOFs to integrate multiple therapeutic modalities. The combination of PDT, NO release, and starvation therapy, along with the homotypic targeting provided by the 4T1 cell membrane, offers a promising approach for the precise and effective treatment of cancer. The successful design of the mPAGT nanosystem underscores the potential of using nMOFs as versatile platforms for multifunctional cancer therapy.

## 13 Biomimetic por-nMOFs for immuno-enhanced PDT

Cancer cell and red blood cell membranes can enhance the circulation stability of nMOFs, help evade phagocytosis by immune cells, and improve targeting to cancer cells (Li et al., 2017b). However, these biological membranes themselves have limited therapeutic effects. Researchers have found that hybrid membranes, such as those obtained by fusing cancer cell membranes with immune cell membranes or co-extrusion, possess enhanced biological functions (Gao et al., 2024). Due to the inherent immunostimulatory, cancer-targeting, and cytotoxic properties, immune cell membranes have become a focus of research for many groups (Gao et al., 2024). For instance, as part of the innate immune barrier, natural killer (NK) cells play a protective role in various inflammatory environments (Gao et al., 2024). Numerous studies have shown that NK cells effectively activate the immune system *in vivo* (Shimasaki et al., 2020). The surface proteins on NK cell membranes can recognize malignant tumor cells and activate phagocytes and other immune cells, thereby exerting immunomodulatory functions and eliminating tumor cells (Myers and Miller, 2021). Therefore, researchers hypothesized that encapsulating nanomaterials with NK cell membranes might stimulate the immunomodulatory functions of the prepared nanosystems.

**FIGURE 12:**
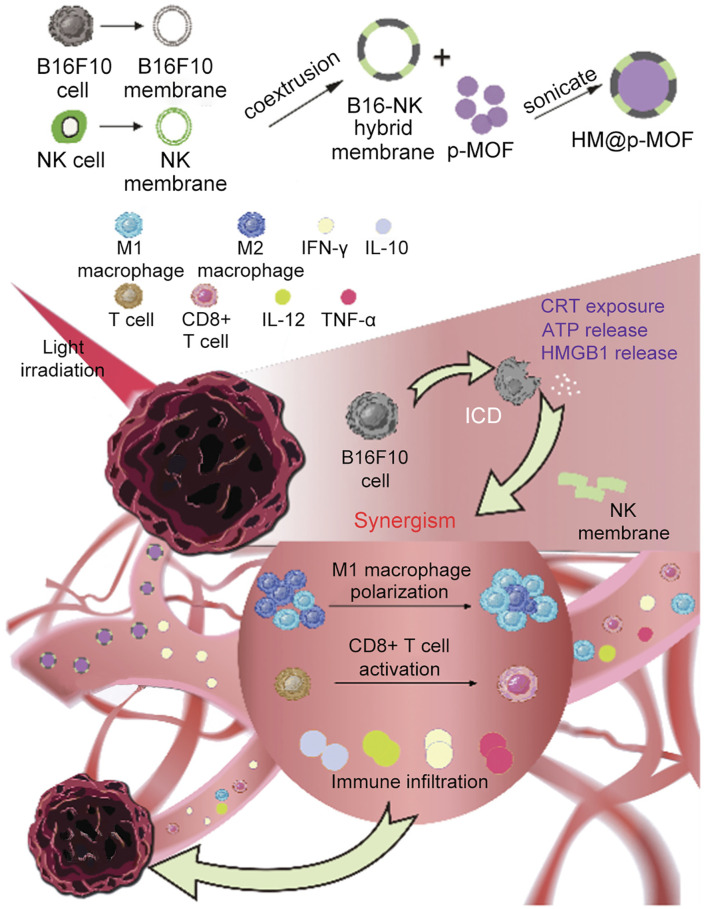
Schematic representation of the HM@p-MOF nanoplatform for immuno-enhanced tumor phototherapy. Reproduced with permission from (Gao et al., 2024). Copyright (2024), Elsevier.

For example, Yu et al. synthesized PCN MOF (p-MOF) using Zr and TCPP. They then coated the p-MOF with a hybrid membrane obtained by co-extruding B16F10 cancer cell and NK immune cell membranes, resulting in the HM@p-MOF nanosystem (Figure 12) (Gao et al., 2024). Subsequent cellular experiments demonstrated that the HM@p-MOF nanosystem, assisted by the hybrid membrane, exhibited strong cancer cell targeting. Moreover, the inherent immune-activating function of the immune cell membrane enhanced the PDT effect induced by the porphyrin photosensitizer under light exposure, leading to immunogenic cell death (Figure 12). *In vivo* experimental results showed that the HM@p-MOF + L group had the most potent antitumor effect on B16F10 cancer cells compared to various control groups. Subsequently, they conducted bilateral tumor model experiments and measured the volume changes of metastatic tumors and the survival rates of mice in different treatment groups. Results showed that the HM@p-MOF + L group had the smallest metastatic tumor volumes and the longest mouse survival times. These experimental results are consistent with the expected outcomes, indicating that the enhanced ICD effect from the hybrid membrane structure can trigger systemic immune responses in mice.

## 14 Biomimetic por-nMOFs for combined PDT + CDT + ferroptosis + imunotherapy

In addition to the co-extrusion of NK cell membranes with cancer cell membranes to create hybrid membranes that possess both immunomodulatory and cancer-targeting properties, researchers have found that dendritic cells (DCs) can also be fused with cancer cells (Cai et al., 2024). The resulting fusion cell membranes not only effectively encapsulate and deliver nanomaterials but also simultaneously target cancer cells and activate immune responses (Cai et al., 2024). DC membranes can enable the expression of complete antigens on the surface of the hybrid membranes, thereby achieving optimal immunotherapeutic effects. Immunotherapy is extensively studied and holds significant potential in cancer treatment (Chen and Wang, 2020; Hu et al., 2020). DC membranes-enhanced immune function has been a particularly promising approach for activating the immune system.

From this, Cai et al. used a self-templating strategy to mix an ethanol solution containing TCPP, oridonin (ORI), and CaO_2_ with an ethanol solution of Fe^3+^ ions (Cai et al., 2024). After stirring and centrifugation, they obtained por-nMOFs NPs loaded with oridonin and CaO_2_. Subsequently, the authors prepared a fusion membrane from DC and B16F10 cancer cell membranes and mixed it with the NPs via ultrasonication, resulting in the fusion membrane-coated nanosystem FM@NPs. At the cellular level, the authors demonstrated the multifunctional therapeutic effects of the prepared nanosystem. The fusion membrane coating facilitated the internalization of the nanomedicine by cancer cells. Inside the cells, the Fe^3+^ in the NPs interacted with excess intracellular GSH, forming Fe^2+^ and reducing the levels of reductive substances (Wu et al., 2024). Meanwhile, under mildly acidic conditions, CaO_2_ generated H_2_O_2_ and oxygen. The produced O_2_ alleviated tumor hypoxia, which is beneficial for PDT. Additionally, the excess Ca^2+^ made cancer cells more sensitive. Furthermore, the H_2_O_2_ was generated. Along with the existing intracellular H_2_O_2_, it reacted with the formed Fe^2+^ through a chemodynamic mechanism and produced highly oxidative ·OH. This led to significant lipid peroxidation, and the reduction in GSH concentration ultimately induced ferroptosis. On the other hand, the acidic environment caused the degradation of the nMOFs framework, and the interaction between Fe^3+^ ions and GSH promoted the release of ORI molecules. The released ORI molecules effectively inhibited both the HSPB1/PCBP1/IREB2 and FSP1/COQ10 pathways, further inducing ferroptosis. Moreover, the immunomodulatory effect of the fusion membrane enabled the nanomedicine to effectively inhibit melanoma proliferation. This system achieved a multifunctional combined therapeutic effect, enhanced by the cancer-targeting properties of the fusion membrane. These combined mechanisms included the PDT function of the porphyrin photosensitizer, Fe^3+^/Fe^2+^-mediated CDT, ORI-mediated ferroptosis, and the immune-stimulating effect promoted by the fusion membrane. The synergistic interactions among these therapeutic effects significantly suppressed melanoma proliferation. The rational design of this system promotes the antitumor application of por-nMOFs and provides a new strategy for the treatment of highly malignant tumors such as melanoma.

## 15 Comparison of por-nMOFs encapsulated with different cell membranes for enhanced PDT

**FIGURE 13:**
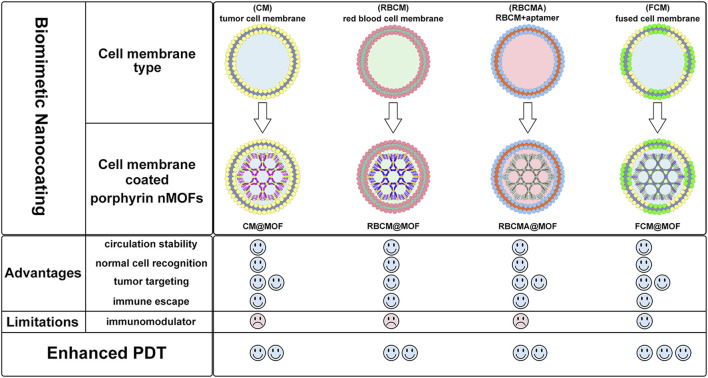
Comparison about the advantages and limitations of different cell membranes and their combination with porphyrin nMOFs for PDT application.

The framework structure of nMOFs endows porphyrin photosensitizers with unique advantages, such as inhibiting molecular aggregation and enhancing the quantum yield of ^1^O_2_ (Lu et al., 2014). However, for por-nMOFs to exert their therapeutic functions after intravenous injection, they must undergo several processes common to nanomedicines (Sun et al., 2014). These processes include circulation in the bloodstream, accumulation within the tumor, penetration into the tumor tissue, and subsequent internalization by cancer cells, followed by the generation of ^1^O_2_ under light exposure for PDT. Although nMOFs generally exhibit good biocompatibility, most nMOFs carry a positive surface charge in physiological solutions, which is not conducive to long-term circulation in the blood. Researchers have found that the zeta potential of most cell membranes is below zero (Gao et al., 2013; Kroll et al., 2017a). When combined with nMOFs, this results in a negative surface charge on the prepared nanosystems, which is favorable for prolonged circulation in the blood. Both cancer cell membranes and red blood cell membranes can enhance the dispersibility and stability of nMOFs (Figure 13).

Furthermore, summarizing the relevant experimental results, it is evident that despite the strong affinity between proliferating cancer cells. This allows them to aggregate and form solid tumors, and this unique characteristic is due to the complex antigen profile on the cancer cell surface (Fang et al., 2017). Cancer cell membrane-coated MOFs (CM@MOFs) inherit these properties. Through the recognition of surface proteins, CM@MOFs exhibit significant homotypic targeting capabilities. In contrast, red blood cell membranes do not contain cancer-specific targeting molecules and thus cannot actively target tumors (Figure 13) (Gao et al., 2013).

Additionally, cancer cells not only possess robust survival and dissemination abilities but can also mimic certain signaling pathways using their complex membrane components, thereby evading immune surveillance and achieving immune tolerance (Fang et al., 2018). Cell membrane-coated nanosystems can effectively reduce phagocytic clearance, leading to better long-term circulation and ultimately maximizing accumulation in tumor cells (Kroll et al., 2017b). Red blood cell membranes also contain CD47 receptors, which inhibit macrophage phagocytosis of red blood cells, further aiding in the long-term circulation of the nanosystem in the blood (Gao et al., 2013). Experimental results show that both cancer cell and red blood cell membrane-coated nanoparticles result in similarly low phagocytic uptake, indicating comparable immune evasion capabilities (Figure 13). Over the past few decades, extensive research has been focused on utilizing polyethylene glycol (PEG)-based polymeric materials to modify nanomedicines, aiming to evade the immune clearance within organisms and prolong the blood circulation time (Rao et al., 2015). However, it is widely recognized that PEG polymers can trigger skin allergies. Additionally, the literature indicates that upon the second injection, PEG-modified nanoplatforms might be rapidly eliminated *in vivo* (Rao et al., 2015). To enable nanomedicines to more effectively evade immune clearance and achieve a safer and longer blood circulation duration, researchers have identified that membrane materials like red blood cell membranes are highly promising alternatives (Miao et al., 2022). A large number of preclinical studies have verified the outstanding properties of nanoplatforms encapsulated by red blood cell membranes. For instance, Rao et al. have shown that Fe_3_O_4_ enwrapped with red blood cell membranes exhibits a significantly prolonged blood circulation time and can effectively resist phagocytosis by phagocytes (Rao et al., 2015). Bidkar et al. have utilized red blood cell membranes to encapsulate doxorubicin and methylene blue, capitalizing on the extended circulation time of the cell membranes to achieve more effective combined chemotherapy and photodynamic therapy (Bidkar et al., 2020). Zhang et al. have also demonstrated that the nanosystem coated with red blood cell membranes can effectively circumvent the immune clearance of organisms, thereby prolonging the blood circulation time (Miao et al., 2022). Collectively, these studies have convincingly demonstrated that cell membrane coating holds great significance for enhancing the blood circulation stability of nanomedicines and realizing the long-term circulation drug-delivery mechanism.

Beyond the use of individual cancer cell and red blood cell membranes, some researchers have combined cancer cell membranes with immune cell membranes to impart immunomodulatory functions to the hybrid membranes (Yu et al., 2024). Immune cell membranes, including those from natural killer (NK) cells and dendritic cells (DCs), are rich in antigens that can recognize tumor cells and activate phagocytes (Cai et al., 2024; Gao et al., 2024). NK cells can also secrete interferons and tumor necrosis factors, promoting the maturation and activation of DCs and modulating immune responses (Gao et al., 2024). DC membranes, when fused with cancer cell membranes, can enhance the immunomodulatory function of the hybrid membranes (Cai et al., 2024). This hybrid fusion approach leverages the complete tumor antigen presentation by immune cell membranes, significantly enhancing the therapeutic effects of PDT-induced immunogenic cell death and suppressing metastatic tumors through systemic immune activation (Figure 13). Combining cell membranes with nMOFs represents a top-down strategy. This approach not only improves the dispersibility and stability of nMOFs, promotes immune evasion, and achieves cancer cell targeting, but also enhances the therapeutic efficacy of PDT-induced ICD by incorporating immune cell membranes (Gao et al., 2024). This integrated design provides an effective solution for the multifunctional application of nMOFs and points to new directions for the phototherapeutic applications of porphyrin-based photosensitizers.

## 16 Conclusion and perspectives

The advancement of nanotechnology has witnessed rapid development in the application of por-nMOFs for phototherapy and other combined therapeutic approaches against tumors (Liu et al., 2016). This review provides a comprehensive summary of the phototherapeutic applications of por-nMOFs encapsulated by cancer cell membranes, red blood cell membranes, aptamer-modified red blood cell membranes, and hybrid membranes derived from the fusion of cancer and immune cell membranes. By carefully controlling the feed ratios, catalyst amounts, and reaction times, nMOFs with large specific surface areas can be synthesized, providing a foundation for various functional applications that require surface modifications (Park et al., 2016). The effective co-extrusion strategy allows for the integration of cell membranes with nMOFs (Li et al., 2017a). These biological structures, evolved over millions of years, possess unique advantages that cannot be replicated by synthetic materials. Cancer cell-derived membranes retain a diverse range of antigens and biological functions of the original cancer cells. When combined with por-nMOFs nanosystems, these membranes confer significantly enhanced performance, including improved dispersion stability, circulatory stability, evasion of phagocytic clearance, and selective homotypic targeting of cancer cells while sparing normal cells. Moreover, if the hybrid membranes are formed from the fusion of immune and cancer cells, they inherently carry immunomodulatory properties that can augment the immunogenic cell death induced by PDT, activate systemic immunity, and inhibit cancer metastasis (Yu et al., 2024). The inspiration and application of biomimetic membranes highlight the vast potential of nMOFs when functionalized for antitumor applications. Cell membrane-coated por-nMOFs represent a nearly ideal nanomedicine, offering new hope for the deeper exploration of porphyrin photosensitizers in cancer therapy.

Despite the evident advantages of cell membrane-assisted nanomedicines, several challenges must be addressed to facilitate their widespread application (Liu et al., 2020). First, the extraction of cell membranes is time-consuming and costly, and simple extraction methods do not always ensure that all extracted membranes contain the desired functional surface antigens. Second, there is a need to explore novel cell membrane preparation techniques. For instance, various membrane extraction methods and optimization of conditions should be explored to obtain a greater quantity of high-quality cell membranes for large-scale production. Third, as delicate biological structures, the long-term storage stability of cell membranes may limit their use as a scalable material for nanomedicine preparation. Fourth, sophisticated instruments are required to closely monitor structural changes after the cell membrane coating process, as the orientation of the inner and outer leaflets of the membrane can influence its biological function. Fifth, the safety of biologics is paramount, and it is crucial to thoroughly verify that genetic material from cancer cells is completely removed during the membrane extraction process to avoid introducing new pathogenic factors. Sixth, to comprehensively assess the safety of nanomedicines coated with cell membranes, a broader range of cancer types should be considered, including various subtypes of solid tumors (such as pancreatic cancer and liver cancer) and hematological malignancies (such as leukemia). By conducting experiments in these diverse cancer models, the therapeutic effects and potential side effects of the system can be evaluated more thoroughly, and its applicability and advantages in different cancer environments can be further identified. Seventh, to address the issue of membrane stability, it is necessary to conduct in-depth research on the factors affecting cell membrane stability, such as the complex physiological environment *in vivo* (changes in pH value, the action of enzymes, etc.). On the one hand, physical or chemical modifications of cell membranes should be attempted, such as cross-linking treatment or the addition of specific stabilizers. On the other hand, the combination method of nanocarriers and cell membranes should be optimized to develop more stable connection strategies, ensuring that cell membranes maintain a stable structure and function throughout the treatment process, thereby guaranteeing the therapeutic potential of the system. Concurrently, the biological effects of nMOFs with varying particle sizes after their interaction with cell membranes should be investigated. This is not only because the size of the nanoparticles critically influences their transport behavior *in vivo*, but also because the surface modification that facilitates this interaction is affected by the specific surface area (Xu et al., 2023). Ultimately, further attempts to combine cell membranes with nMOFs will contribute to a deeper understanding of the advantages and potential drawbacks associated with employing this biomimetic strategy to enhance the properties of nMOFs materials (Krishnan et al., 2023). Addressing these issues will accelerate the development of cell membrane-based nanomedicines. Overall, the combination of biomimetic membranes and por-nMOFs represents a highly promising research direction, which can promote the multifunctional applications of por-nMOFs and lay a solid foundation for their biomedical applications.
